# Clinical Relevance of Gain- and Loss-of-Function Germline Mutations in STAT1: A Systematic Review

**DOI:** 10.3389/fimmu.2021.654406

**Published:** 2021-03-11

**Authors:** Wenjing Zhang, Xuemei Chen, Guodong Gao, Shubin Xing, Lina Zhou, Xuemei Tang, Xiaodong Zhao, Yunfei An

**Affiliations:** ^1^Department of Rheumatology and Immunology, Children's Hospital of Chongqing Medical University, Chongqing, China; ^2^College of Computer and Information Science, Southwest University, Chongqing, China; ^3^Ministry of Education Key Laboratory of Child Development and Disorders, Children's Hospital of Chongqing Medical University, Chongqing, China; ^4^National Clinical Research Center for Child Health and Disorders, Children's Hospital of Chongqing Medical University, Chongqing, China; ^5^China International Science and Technology Cooperation Base of Child Development and Critical Disorders, Children's Hospital of Chongqing Medical University, Chongqing, China; ^6^Chongqing Key Laboratory of Child Infection and Immunity, Children's Hospital of Chongqing Medical University, Chongqing, China

**Keywords:** signal transducer and activator of transcription 1, chronic mucocutaneous candidiasis, Mendelian susceptibility to mycobacterial diseases, GoF, LOF, hypothyroidism

## Abstract

**Background:** Germline mutations in signal transducer and activator of transcription 1 (STAT1), which lead to primary immunodeficiency, are classified as defects in intrinsic and innate immunity. To date, no comprehensive overview comparing GOF with LOF in early-onset immunodeficiency has been compiled.

**Objective:** To collect and systematically review all studies reporting STAT1 GOF and LOF cases, and to describe the clinical, diagnostic, molecular, and therapeutic characteristics of all the conditions.

**Methods:** A systematic review of the PubMed, EMBASE, Web of Science, Scopus, and Cochrane to identify articles published before May 23, 2020. Data pertaining to patients with a genetic diagnosis of STAT1 GOF or LOF germline mutations, along with detailed clinical data, were reviewed.

**Results:** The search identified 108 publications describing 442 unique patients with STAT1 GOF mutations. The patients documented with chronic mucocutaneous candidiasis (CMC; 410/442), lower respiratory tract infections (210/442), and autoimmune thyroid disease (102/442). Th17 cytopenia was identified in 87.8% of those with GOF mutations. Twenty-five patients with GOF mutations received hematopoietic stem cell transplantation (HSCT), and 10 died several months later. Twelve of 20 patients who received JAK inhibitor therapy showed improved symptoms. Twenty-one publications described 39 unique patients with STAT1 LOF mutations. The most common manifestations were Mendelian susceptibility to mycobacterial diseases (MSMD) (29/39), followed by osteomyelitis (16/39), and lymphadenopathy (9/39). Missense, indel, and frameshift mutations were identified as LOF mutations. There were no obvious defects in lymphocyte subsets or immunoglobulin levels. Eighteen patients required antimycobacterial treatment. Three patients received HSCT, and one of the three died from fulminant EBV infection.

**Conclusions:** STAT1 GOF syndrome is a clinical entity to consider when confronted with a patient with early-onset CMC, bacterial respiratory tract infections, or autoimmune thyroid disease as well as Th17 cytopenia and humoral immunodeficiency. HSCT is still not a reasonable therapeutic choice. Immunoglobulin replacement therapy and JAK inhibitors are an attractive alternative. STAT1 LOF deficiency is a more complicated underlying cause of early-onset MSMD, osteomyelitis, respiratory tract infections, and Herpesviridae infection. Anti-mycobacterial treatment is the main therapeutic choice. More trials are needed to assess the utility of HSCT.

## Introduction

Signal transducer and activator of transcription 1 (STAT1) is one of the most important members of the STAT family. STAT1 plays a critical role in regulating cell growth, differentiation, proliferation, metabolism, and apoptosis through the JAK-STAT pathway (1, 2). Somatic mutations in STAT1 have been discussed in the context of cancer for a long time (3). Germline STAT1 LOF mutations with MSMD (Mendelian susceptibility to mycobacterial disease) and STAT1 GOF mutations with CMC (chronic mucocutaneous candidiasis) were first reported in 2001 and 2011, respectively; since then, research has confirmed that both STAT1-GOF and LOF germline mutations cause immunodeficiency and immune dysregulation, with a wide clinical spectrum beyond malignancies (4–6). In 2016, a prospective cohort study of 274 patients with STAT1 GOF mutations was published (7). Patients with heterozygous STAT1 GOF mutations usually present with CMC, although some may also suffer bacterial and viral infections, autoimmune manifestations, lymphopenia, cerebral aneurysms, and increased risk of developing tumors (8). Okada et al. (9) comprehensively reviewed genetics, cellular, molecular and biological, clinical features including CMC and Type I Interferonopathy. More recently, another 168 cases, with new clinical features that overlap those of STAT1 LOF mutations, were reported. In addition, new therapies such as ruxolitinib and baritinib have been reported for CMC or steroid-dependent severe autoimmunity caused by STAT1 GOF (10–12). Homozygous or compound heterozygous mutations of STAT1 lead to complete or partial STAT1 LOF, which is associated with susceptibility to infection by intracellular pathogens and herpetic infections (13–16). No comprehensive overview of STAT1 LOF mutations has been published to date. The phenotype caused by STAT1 mutation is highly diverse, and there is considerable overlap between GOF and LOF phenotypes; thus, clinical definition and diagnosis are difficult. Here, we seek to undertake a systematic review of STAT1 GOF or LOF cases published up until May 2020, and describe the genetic, functional, and clinical manifestations of these mutations.

## Methods

### Patients

This systematic review was conducted in accordance with the Preferred Reporting Items for Systematic Reviews and Meta-analyses (PRISMA) guidelines (PROSPERO registration number: CRD42020192162).

A systematic search of the literature was conducted on December 10, 2019, to identify studies reporting patients with STAT1 GOF germline mutations. Five databases were searched: MEDLINE (PubMed), Web of Science, the Cochrane Central Register of Controlled Trials, EMBASE, and Scopus. The literature search was repeated on May 11, 2020, to update the information. The key search terms were: Keywords CONTAINS “STAT1” or “Signal Transducer and Activator of Transcription 1,” or Title CONTAINS “STAT1” or “Signal Transducer and Activator of Transcription 1” AND Keywords CONTAIN “loss-of-function” or “loss-of-function” or “LOF” or “leaky mutation,” or “leaky mutations” or “null mutation” or “null mutations” or “negative mutation” or “negative mutations” or “gain-of-function” or “gain-of-function” or “GOF” or “activating germline mutation” or “activating germline mutations” or “activating mutation” or “activating mutations” or “immunodeficiency” or “immunodeficiency” or “deficiency,” or Title CONTAINS “loss-of-function” or “loss-of-function” or “LOF” or “leaky mutation” or “leaky mutations” or “null mutation” or “null mutations” or “negative mutation” or “negative mutations” or “gain-of-function” or “gain-of-function” or “GOF” or “activating germline mutation” or “activating germline mutations” or “activating mutation” or “activating mutations” or “immunodeficiency” or “immunodeficiency” or “deficiency.” Another search of Google Scholar was performed manually. Relevant reviews and large case series were checked to identify potentially eligible studies or unpublished data. In some cases, the corresponding authors of the articles were contacted. Abstracts and full text were screened to identify all suitable articles. Duplicate articles or congress abstracts were excluded. To be eligible for inclusion, studies and case reports had to describe patients with STAT1 germline mutations that had undergone genetic analysis; if functional analyses (e.g., immunoblotting or luciferase assays) were not performed to confirm the mutation then a concordant phenotype had to be identified based on clinical manifestations and family history. All types of publication (articles, reviews, editorials, letters, and correspondence), written in English or French, and published online between July 2001 and May 2020 were included.

### Statistical Analysis

Data were collected and organized using Microsoft Excel (Microsoft), and statistical analysis was performed using SPSS 25. Data are presented as the median and (first, third) quartiles. Additional information about data extraction and quality assessment is provided in the Supplementary Materials.

## Results

**Figure 1:**
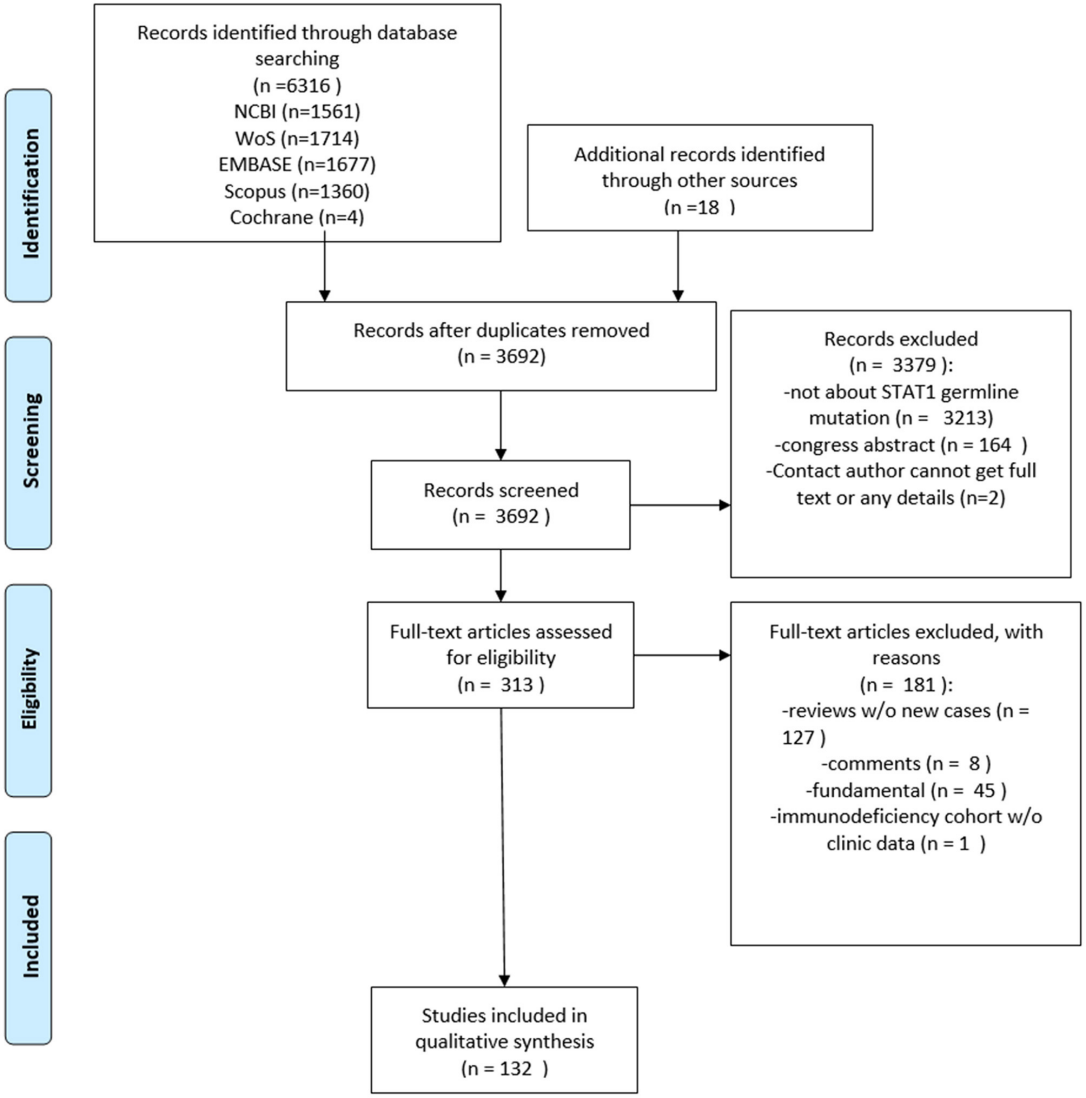
PRISMA flow diagram.

The literature search identified 108 publications describing 442 unique patients with STAT1 GOF mutations and 21 publications describing 39 unique patients with STAT1 LOF mutations. The PRISMA diagram is shown in Figure 1.

### Baseline Characteristics of Patients With STAT1 GOF Mutations

In this study, we evaluated 442 patients (224 males, 209 females, and 9 of unknown gender) with a molecular diagnosis of STAT1 GOF. The GOF mutation in the STAT1 gene was most common in France, the USA, Germany, Japan, the UK, China, and the Netherlands. The median (IQR) age of patients at the time of study was 18.0 (9.0–33.0) years, and that for age of onset was 1.0 (0.5–5.5) years. The median age at the time of diagnosis was 6.2 (3.5–13.5) years. The median (IQR) delay in the diagnosis of STAT1 defects was 5.7 (2.9–12.5) years. The longest diagnostic delay was 25 years following the onset of symptoms, whereas the shortest was 0.8 years (Table 1). Forty-six patients came from families in which multiple individuals were affected. At the time of our analysis, 383 patients (86.7%) were alive (overall survival is shown in Supplementary Figure 1B), whereas 59 (13.3%) were deceased (mostly due to infection or tumors). Sepsis, disseminated fungal or viral infections, hemophagocytic syndrome, hemorrhage (including intracranial, cerebral, and lung hemorrhage), multiple organ failure, pneumonia, pulmonary embolism, hepatitis, pulmonary hypertension, and disseminated intravascular coagulation were recorded as the causes of death in those with STAT1 GOF.

**Table 1:** Demographic data of patients with STAT1 gain-of-function mutation.

**Parameter (no. of evaluated patients)**	**Results**
Country of residence (%) (*n* = 371); NK (*n* = 71)	France (13.7), USA (12.9), Germany (9.7), Japan (8.4), UK (7.8), China (5.1), Netherlands (5.1), Mexico (3.5), Italy (2.7), Canada (2.4), Turkey (2.4), Belgium (2.2), Czech Republic (1.6), Norway (1.6), Switzerland (1.6), Argentina (1.3), Morocco (1.3), Chile (1.1), Hungary (1.1), Iran (1.1), Israel (1.1), Spain (1.1), and Other (11.1)
Ethnicity (%) (*n* = 42)	France (16.7), Germany (14.3), Japan (14.3), Saudi Arabia (12.0), Israel (9.5), Denmark (7.1), and other (28.6)
Sex ratio, M/F, *n* (%) (*n* = 433 and NK = 9)	224 (51.7)/209 (48.3)
Consanguinity, *n* (%) (*n* = 310 and NK = 132)	13 (4.2)
Familial case, *n* (%) (*n* = 374 and NK = 68)	232 (62.0)
Alive/dead, *n* (%) (*n* = 442)	383 (86.7)/59 (13.3)
Age (y) (*n* = 419 and NK = 23)	Min = 0.1, max = 85.0; median (IQR) = 18.0 (9.0–33.0)
Age at onset (y) (*n* = 96 and NK = 346)	Min = 0.0, max = 30.0; median (IQR) = 1.0 (0.5–5.5)
Age at diagnosis (y) (*n* = 15 and NK = 427)	Min = 1.0, max = 26.0; median (IQR) = 6.2 (3.5–13.5)
Delay in diagnosis (y) (*n* = 15 and NK = 427)	Min = 0.8, max = 25.0; median (IQR) = 5.7 (2.9–12.5)
Age at presentation of CMC (y) (*n* = 312 and NK = 130)	Min = 0.0, max = 50.0; median (IQR) = 1.0 (0.3–4.3)

*NK, Not known*.

### Baseline Characteristics of Patients With STAT1 LOF Mutations

Thirty-nine patients (17 males, 17 females, and 5 of unknown gender) with STAT1 LOF mutation were reported; mutation was most common in France, Germany, Japan, and Saudi Arabia (Table 2). The median (IQR) age at the time of study was 5.0 (1.5–13.5) years. Overall survival is shown in Supplementary Figure 1C. The median (IQR) age of onset was 0.7 (0.3–2.0) years, and the median age at the time of diagnosis was 3.0 (2.2–12.5) years. The median (IQR) delay in diagnosis of immunodeficiency was 2.7 (1.0–9.9) years. The longest diagnostic delay was 32 years following onset of symptoms, whereas the shortest was 0.7 years. Twenty-five patients came from families in which multiple individuals were affected. At the time of our analysis, 35 patients (82.1%) were alive, and seven (17.9%) were deceased, mostly due to viral infections.

**Table 2:** Demographic data of patients with STAT1 LOF immunodeficiency.

**Parameters (no. of evaluated patients)**	**Results**
Country of living (%) (*n* = 39)	France (17.9), Germany (15.4), Japan (15.4), Saudi Arabia (12.8), Israel (10.3), Denmark (7.7), and other (20.5)
Ethnicity (%) (*n* = 39)	France (17.9), Germany (15.4), Japan (15.4), Saudi Arabia (12.8), Israel (10.3), Denmark (7.7), and other (20.5)
Sex ratio, M/F, *n* (%) (*n* = 34 and NK = 5)	17(50)/17(50)
Consanguinity, *n* (%) (*n* = 23 and NK = 16)	10(52)
Familial case, *n* (%) (*n* = 28 and NK = 11)	25(89)
Alive/dead, *n* (%) (*n* = 39)	35(83)/7(17)
Age (y) (*n* = 31 and NK = 8)	Min = 0.3, max = 49.0, median (IQR) = 5.0 (1.5–13.5)
Age at onset (y) (*n* = 25 and NK = 6; no symptoms, *n* = 8)	Min = 0.0(0.02, max = 18.0, median (IQR) = 0.7 (0.3–2.0)
Age at diagnosis (y) (*n* = 10 and NK = 29)	Min = 0.9, max = 33.0; median (IQR) = 3.0 (2.2–12.5)
Delay in diagnosis (y) (*n* = 10 and NK = 29)	Min = 0.7, max = 32.0; median (IQR) = 2.7(1.0–9.9)
Age at presentation of MSMD (y) (*n* = 23 and NK = 2; without presentation (*n* = 14)	Min = 0.2, max = 18.0; median (IQR) = 1.3 (0.6–7.0)

### Genetics and Functional Analysis

Functional studies were reported in 81 articles, and confirmed GOF or LOF of STAT1. The functional tests were as follows:

1. Subcellular distribution or level of STAT-1, STAT-1 tyrosine 701 phosphorylation, ISGF3, and GAF under basal or stimulated conditions (IFN-γ, IFN-α, or IL-27) in SV40 fibroblasts or mouse fibroblast cell lines transfected with wild-type or human STAT1 alleles, or with an insert-less vector or in Epstein–Barr virus-transformed patient cells.
2. Tyrosine 701 phosphorylation levels under basal or stimulated conditions (IFN-α or IFN-γ) in Epstein–Barr virus-transformed patient cells.
3. Gene transcription induced by IFN-α or IFN-γ, as assessed by Northern blotting.
4. *In vitro* STAT1 reporter luciferase assay of STAT1-deficient U3C cells under basal or stimulated conditions (IFN-α or IFN-γ).

### Infections in Patients With STAT1 GOF Mutations

**Figure 2:**
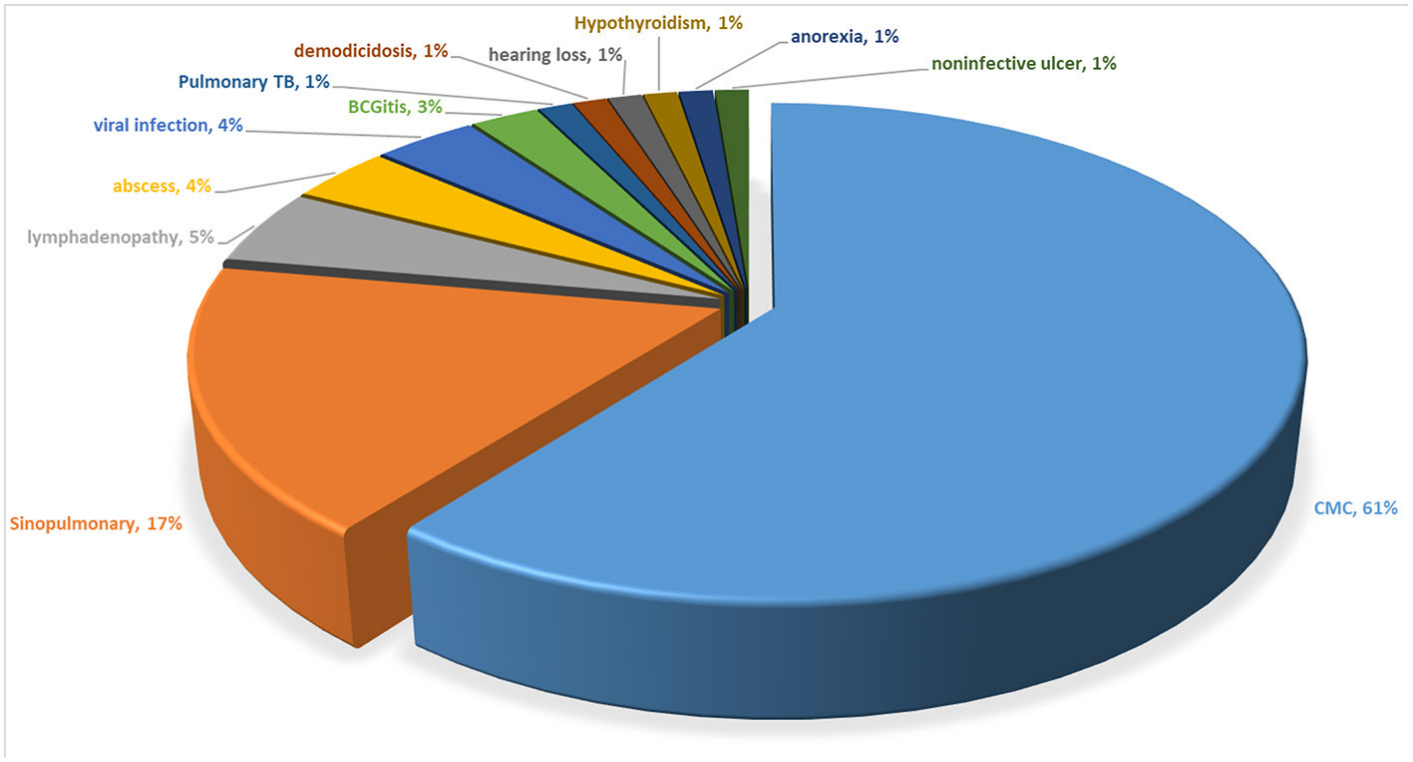
Initial clinical presentation of patients with a STAT1 GOF mutation.

Fungal infections were the most common characteristic. A high proportion of patients presented initially with CMC (60.5%) or a sinopulmonary infection (17.3%) (Figure 2). The median (IQR) age of onset for CMC was 1.0 (0.3-4.3) years, constant with previous results (7). CMC was documented in 410 (92.8%) of the 442 individuals carrying STAT1 GOF mutations. Approximately 90% developed CMC before the age of 10 years (Supplementary Figure 1A). Mucocutaneous fungal infections affected the oral mucosae (75.8%), skin (43.2%), nails (42.8%), esophageal/genital areas (42.1%), and scalp (12.7%). Aphthous stomatitis and invasive fungal infections were also quite common in 138 (31.2%) and 49 (11.1%) patients, respectively (Supplementary Table). *Candida albicans* was the species isolated most frequently (85.7%) from those with mucocutaneous fungal infections but accounted for only a small proportion (18.2%) of invasive infections (Supplementary Table). Twelve patients suffered from invasive *Cryptococcus spp*., 11 from invasive *Pneumocystis jirovecii*, 11 from invasive *Aspergillus spp*., and five from invasive *Penicillium marneffei*. *Penicillium marneffei* was not discussed in Toubiana et al.'s (7) work.

**Figure 3:**
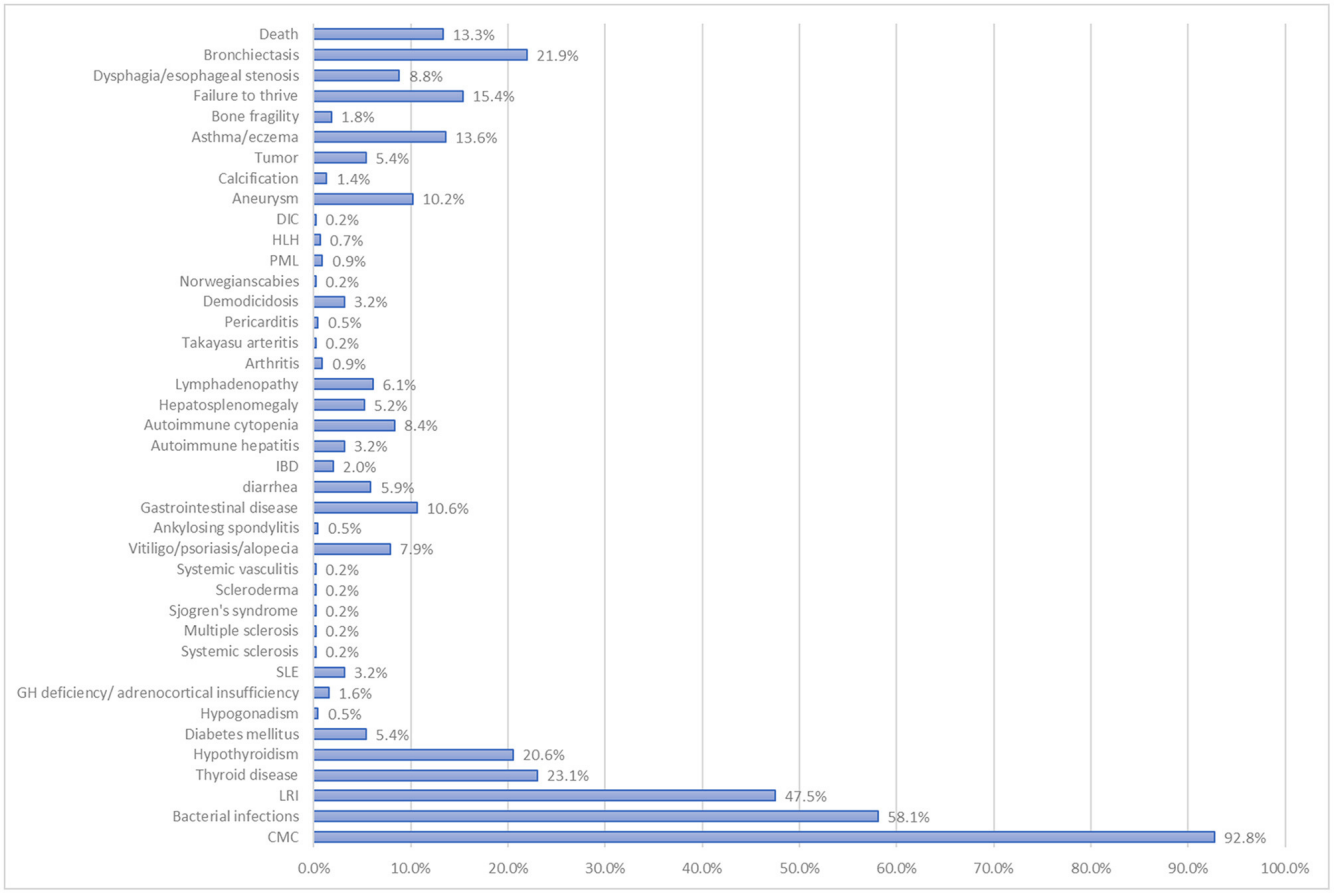
Percentage of patients with the STAT1 GOF mutation that suffer clinical complications.

Patients with STAT1 GOF are also prone to bacterial infections; indeed, these were reported in 257 of the 442 patients. LRIs were reported in 164 patients (37.1%), often with recurrent lobar pneumonia, bronchitis, and/or interstitial pneumonia (Figure 3). *Staphylococcus aureus* (*n* = 55), *Streptococcus spp*. (*n* = 32), *Pseudomonas aeruginosa* (*n* = 22), and *Haemophilus influenzae* (*n* = 16) were the most common pathogens (Supplementary Table). ENT infections were reported in 150 of the 442 patients (33.9%), mostly as recurrent or chronic sinusitis or otitis media. Forty-one patients (9.3%, higher than that of Toubiana et al.'s study) had mycobacterial disease. Lung infections were caused by *Mycobacterium tuberculosis* (*M. tuberculosis, n* = 10) or nontuberculous environmental mycobacteria (*n* = 6). Skin diseases and adenitis were caused by the Bacille Calmette-Guerin (BCG) vaccine or environmental mycobacteria (*n* = 13), and disseminated disease was caused by BCG (*n* = 5), *M. tuberculosis* (*n* = 6), *M. avium* (*n* = 1), or *M. genavense* (*n* = 1) (Supplementary Table).

Among the 216 of 442 patients with documented viral infections, Herpes simplex, Varicella-zoster, CMV, or EBV, and even Molluscum contagiosum/warts were common pathogens (Supplementary Table). Of note, a recent report revealed that four patients with GOF developed progressive multifocal leukoencephalopathy caused by JC polyomavirus; the virus was also found in individuals with lymphoma, chronic lymphocytic leukemia, and/or AIDS (17, 18). Demodicidosis or rosacea were reported in some patients (*n* = 5) (19–21). One patient suffered from Norwegian scabies (22). The JC virus, Demodicidosis, Norwegian scabies were not recorded in previous review (7).

### Other Manifestations in Patients With STAT1 GOF Mutations

**Figure 4:**
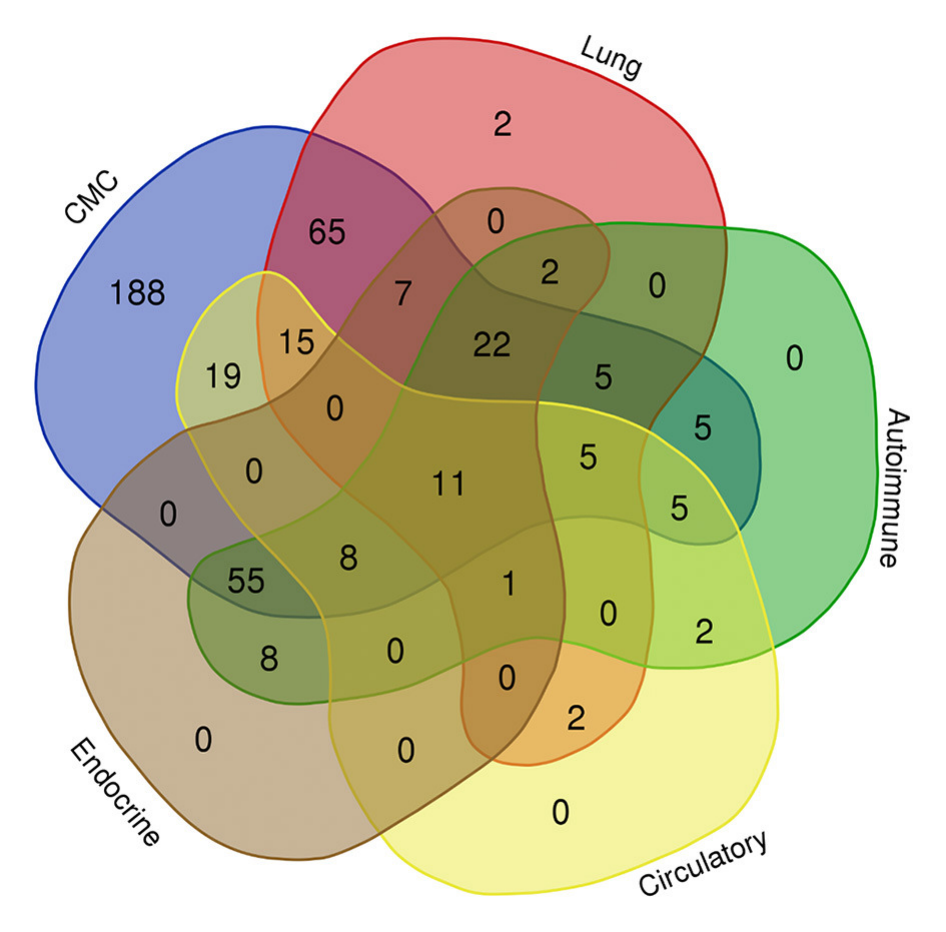
Venn diagram showing common manifestations in different body systems in patients with the STAT1 GOF mutation.

Autoimmune and inflammatory diseases are another characteristic of STAT1 GOF mutations. Indeed, we found that 272 [61.5%, 1.4 times than previous report (7)] of 442 patients displayed clinical autoimmune manifestations, and autoantibodies were documented in at least 120 patients. In addition, 116 (26.2%) patients had CMC accompanied by autoimmune manifestations (Figure 4). Thyroid disease (*n* = 102; 91 of which had hypothyroidism) was the most common, followed by autoimmune cytopenia (*n* = 37), vitiligo/psoriasis/alopecia (*n* = 35), diabetes mellitus (*n* = 24), SLE (*n* = 14), autoimmune hepatitis (*n* = 14), inflammatory bowel disease (*n* = 9), and hypogonadism/GH deficiency/adrenocortical insufficiency (*n* = 9) (Figure 3). Ten had IPEX-like syndrome (23, 24).

The incidence of aneurysm [*n* = 45/442, 10.2%, 1.7 times than previous report (7)] was three times higher than in a control population (3.2%) (25) (Figure 3). Cerebral imaging detected asymptomatic aneurysms in 25 patients, whereas 19% of symptomatic patients suffered hemorrhages. Others had abdominal pain and neurological manifestations, such as hemiplegia, seizures, or attention lapses. The majority of aneurysms were found in the cerebral vascular system (*n* = 16). Extracerebral aneurysms were found in the abdominal and/or thoracic aorta, iliac arteries, and lung arteries (*n* = 4).

Twenty-one patients had cutaneous, oral, laryngeal, esophageal, or gastrointestinal carcinoma, 18 of which were squamous cell carcinomas. One patient had melanoma, two patients had lymphoma, one had acute leukemia, one had prostate cancer plus tongue squamous cell carcinoma, and one had papillary thyroid cancer plus squamous skin cell carcinoma; thus, the overall cancer rate was 5.9% [95% confidence interval, 3.7–8.1, in line with previous report (7)]. Asthma, eczema, and signs of allergy were observed in 60 patients (13.6%). Bone fragility (i.e., osteopenia, osteoporosis, and fracture) was reported in eight patients. The prognosis for those with STAT1 GOF germline mutation was not very good; complications included bronchiectasis (*n* = 97), failure to thrive (*n* = 68), death (*n* = 59), an (d)d dysphagia/esophageal stenosis (*n* = 39) (Figure 3).

### Treatment and Outcome of Patients With STAT1 GOF Mutations

Three hundred and twenty-eight of four hundred and forty-two [74.2% constant with previous report (7)] patients required antifungal treatment; most required two or more fungal drugs due to drug resistance. Only eight patients required topical treatment alone. Fluconazole was the most common first-line treatment, followed by itraconazole or posaconazole. One patient was resistant to nystatin and fluconazole, and partially resistant to voriconazole. He also received IV caspafungin. Finally, the infection was controlled by posaconazole and amphotericin lozenges (26). Data show that antibiotics, mainly co-trimoxazole and macrolides, were used frequently for prophylaxis (in 75/442 vs. 11/442 cases, respectively). Overall, 14% of patients received polyvalent immunoglobulins to treat infections. Despite adequate trough IgG levels after monthly administration of IVIG, some continued to suffer from recurrent lower respiratory tract and/or gastrointestinal tract infections, including Salmonella gastroenteritis (11, 26–29).

Twenty-five [five times than previous report (7)] patients received hematopoietic stem cell transplantation (Table 3); of these, 11 died several months after HSCT and one died from pneumonia and sepsis 2 years post-HSCT. Of the 11 that died, five died from infections (including pneumonia and/or sepsis). HLH, multiorgan failure, and interstitial lung disease were also documented as causes of death. However, another seven patients showed resolution of disease-related symptoms, and four showed immune reconstitution. One patient diagnosed with common variable immune deficiency received HSCT from a matched unrelated donor and achieved full donor chimerism; however, they suffered respiratory symptoms for 552 days during follow-up (29). Pre-conditioning regimen included fludarabine(11/21), ATG(7/21), cyclophosphamide(6/21), treosulfan(5/21), busulfan(5/21), melphalan(4/21), alemtuzumab(4/21). Different from previous report we reviewed the small molecular drugs of JAK inhibitors therapy. Of the STAT1 GOF patients who received JAK inhibitor therapy, 12 of 20 showed improvement of symptoms. One patient receiving oral ruxolitinib (5 mg/m^2^/day) recovered from severe steroid-dependent autoimmunity without side effects and stopped taking steroids (12). In another patient, immunodysregulatory features improved after JAK inhibitor treatment; however, at the same time, the patient suffered lower respiratory tract infection and herpes zoster. He did eventually recover completely and was symptom-free at 15 months post-HSCT (11).

**Table 3:** Treatment of patients with STAT1 GOF mutations.

**Treatment**	**Patients**
Antifungal treatment	*n* = 328
Local treatment only	*n* = 8
Fluconazole	*n* = 183
Posaconazole/itraconazole	*n* = 76
Voriconazole	*n* = 26
Echinocandins	*n* = 6
Terbinafine	*n* = 4
Amphotericin B	*n* = 12
Caspofungin	*n* = 2
Antibiotic prophylaxis	*n* = 75
Co-trimoxazole	*n* = 53
Macrolides	*n* = 25
Antiviral prophylaxis	*n* = 11
Polyvalent immunoglobulins	*n* = 62
Immunotherapy	*n* = 35
Jakinib	*n* = 19
Immunosuppressive therapies	*n* = 26
Hematopoietic stem cell transplantation (HSCT)	*n* = 25

### Infections in Patients With STAT1 LOF Mutations

**Figure 5:**
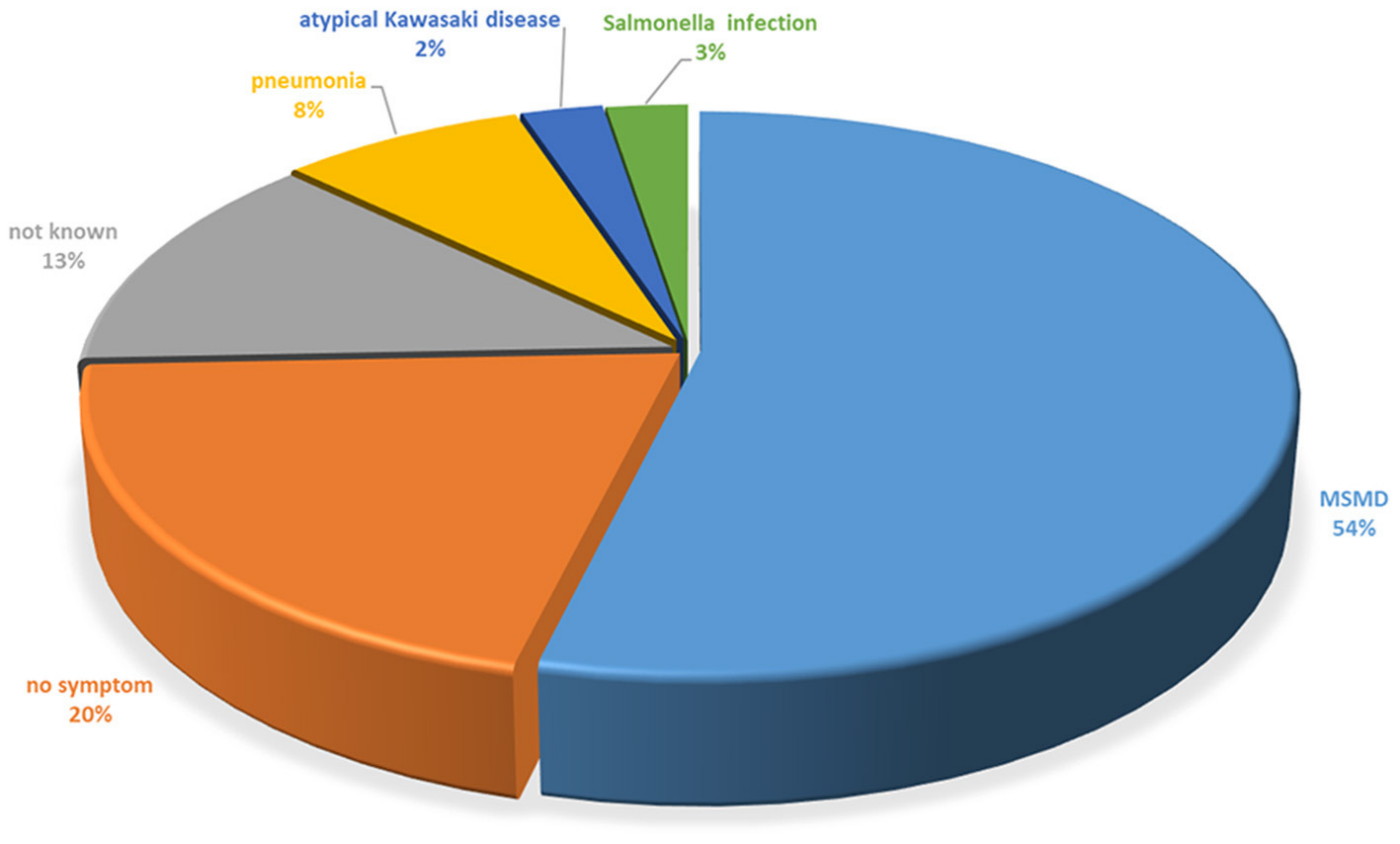
Initial clinical presentation of patients with the STAT1 LOF mutation.

MSMD (54.0%) accounted for most onset symptoms in patients with STAT1 LOF, whereas 3.0% had Salmonella infection (Figure 5). MSMD was documented in 29 (74.4%) of the 39 individuals carrying STAT1 LOF mutations. BCG strain was the most common cause of the first presentation of MSMD (Supplementary Figure 2). The median (IQR) age of MSMD onset was 1.3 (0.6–7.0) years (Table 2). Mycobacteria were the most common cause of disseminated disease (79.2%), bone disease (62.5%), adenitis/skin disease (58.3%), lung disease (16.7%), liver/spleen disease (16.7%), and brain disease (8.3%) (Supplementary Table). Multifocal osteomyelitis was found in one patient, involving the thoracic-lumbar spine, sacrum, left parietal bone of the skull, the right clavicle, the right acetabulum, and one of the middle right ribs; these lesions were accompanied by intestinal tuberculosis and meningitis, which was suspected to be caused by *M. szulgai*. No mycobacteria were cultured from cerebrospinal fluid; however, antimycobacterial therapy resolved the neurological symptoms (30). BCG strain (60.7%) was the most common causative pathogen of MSMD in those with STAT1 LOF. *M. avium* was isolated from five patients (17.9%), as was *M. tuberculosis* (7.1%), *M. szulgai* (3.6%), and *M. kansasii* (3.6%) (Supplementary Table). In some patients, no bacteria were found by PCR sequencing, isolation, or culture. One case was suspected to be sarcoidosis, which was treated with glucocorticoids, although the symptoms progressed gradually (31).

Fourteen patients with STAT1 LOF mutations suffered virus infections, which included EBV (50.0%), VZV (41.7%), HSV (41.7%), CMV (41.7%), HHV (33.3%), RSV (33.3%), adenovirus (25.0%), Molluscum contagiosum/warts (16.7%), and parvovirus (8.3%) (Supplementary Table). Furthermore, all patients suffered systematic viral infections (100%). Bacteremia was recorded in three of five patients with a bacterial infection. Two patients were confirmed salmonella infection from blood or cerebrospinal fluid culture. One had Salmonella group D and B, the other had Salmonella group D. Fungal infections were relatively rare; one patient suffered from *Candida parapsillosis* sepsis resulting from central venous line contamination; this patient also had IgG antibodies against *Toxoplasma gondii* (32). *Aspergillus fumigatus* antigen was identified in the blood of another patient, but no infection was proven (15).

### Other Manifestations in Patients With STAT1 LOF Mutations

**Figure 6:**
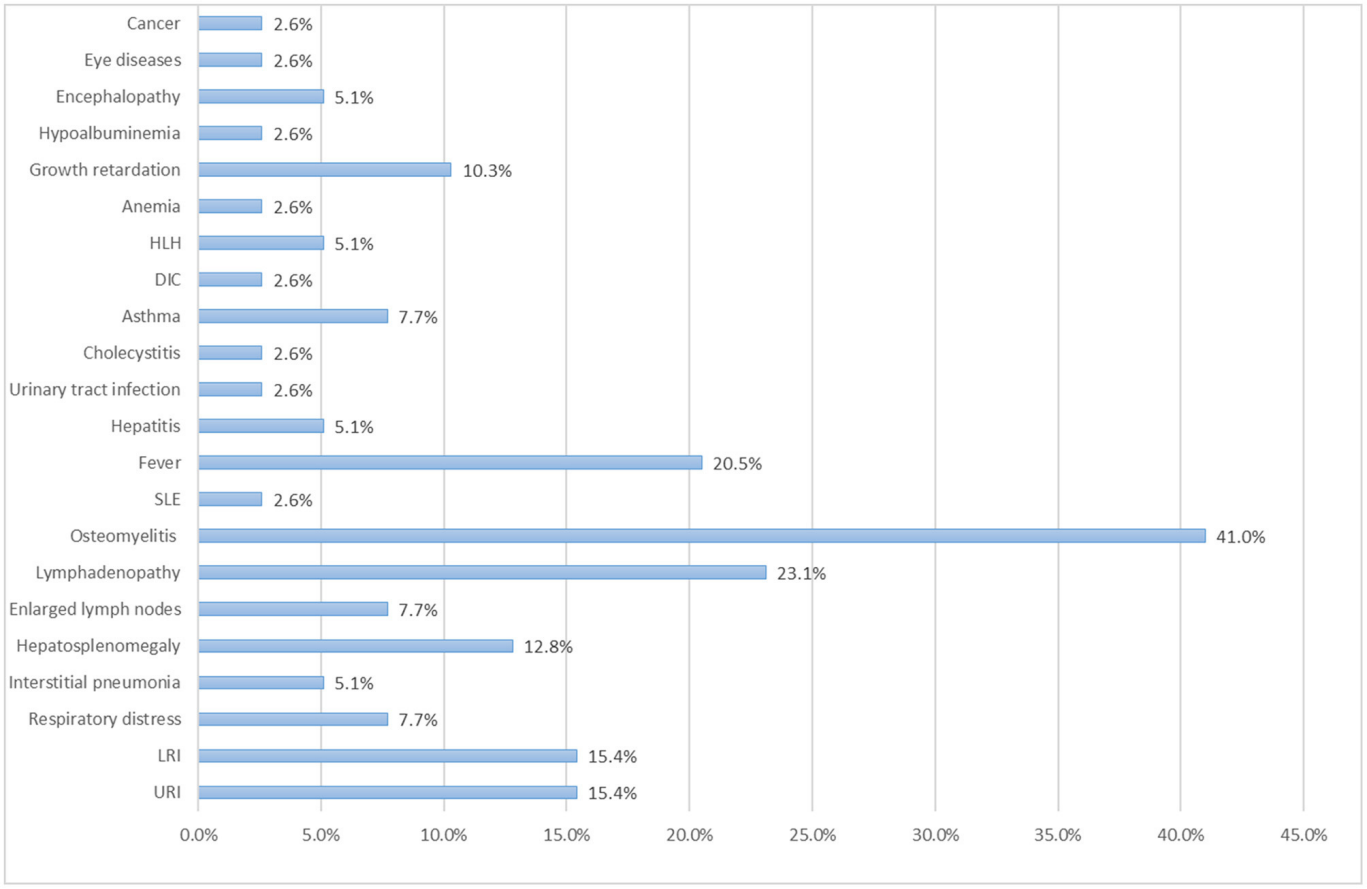
Percentage of patients with the STAT1 LOF mutation that suffer clinical complications.

Osteomyelitis was relatively common in patients with STAT LOF mutations [16/39 (41.0%)] (Figure 6); most cases were caused by mycobacteria. Most patients had multiple lesions, and several were misdiagnosed and received inappropriate treatment. In one child with disseminated BCG osteomyelitis, bone lesions on CT led to an incorrect diagnosis of metastatic neuroblastoma, which was treated with a first cycle of chemotherapy. Another case with multifocal osteomyelitis was suspected to be sarcoidosis, despite histological analysis. Such cases highlight the need to be aware of immune deficiency in patients with multifocal osteomyelitis, especially in those with a poor response to conventional antibiotics. The rate of lymphadenopathy (23.1%) was similar to that of fever (20.5%); quite a few patients showed lymph node enlargement after BCG vaccination. Upper and/or lower respiratory infection was common in those with STAT1 LOF. Three patients suffered respiratory distress and required assisted ventilation. Two patients were diagnosed with interstitial pneumonia. Hepatosplenomegaly occurred in 12.8% of cases, growth retardation in 10.3%, asthma in 7.7%, and hepatitis in 5.1% (Figure 6). Autoimmunity was much less common than in those with STAT1 GOF; only one patient had SLE. One had anemia but without evidence of hemolysis. One suffered two episodes of HLH related to severe infection in 8 and 13 months, respectively; the other had HLH triggered by infection.

### Treatment and Outcome of LOF Patients

Eighteen of 39 patients required antimycobacterial treatment. Seventeen required two, three, or even more drugs to fight the mycobacteria (Table 4). Nine of 18 patients remained symptomatic. Only two of 39 received polyvalent immunoglobulins, whereas eight received biological agents, including IFN (*n* = 6), infliximab (*n* = 1), and rituximab (*n* = 1). For prophylaxis, five were treated with antiviral drugs and four were prescribed Co-SMZ or macrolides. Three patients received HSCT, of these, one died from fulminant EBV infection several months after HSCT, one was weaning off prednisolone 4 months after HSCT, and one had no severe infections during a 9 year follow-up.

**Table 4:** Treatment of patients with STAT1 LOF mutations.

**Treatment**	**Patients**
Antimycobacterial treatment	*n* = 18
Isoniazide	*n* = 11
Rifampicin	*n* = 17
Ethambutol	*n* = 12
Pyrazinamide	*n* = 1
Amikacin	*n* = 2
Fluoroquinolones	*n* = 6
Linezolid	*n* = 2
Antibiotic prophylaxis	*n* = 4
Co-trimoxazole	*n* = 2
Macrolides	*n* = 2
Antiviral prophylaxis	*n* = 5
Polyvalent immunoglobulins	*n* = 2
Immunotherapy	*n* = 8
IFN	*n* = 6
Infliximab	*n* = 1
Rituximab	*n* = 1
Immunosuppressive therapies	*n* = 4
Hematopoietic stem cell transplantation (HSCT)	*n* = 3

### Immunological Investigation of Patients With STAT1 GOF Mutations

Among the 294 STAT1-GOF patients for whom data were available, 67 of 291 patients (23%) for whom T cell data were available had low total T cell frequency (Supplementary Table). T cell subsets analysis revealed reduced CD4+ and CD8+ T cells in 85 (30.8%) and 48 (17.9%) patients, respectively, while 10 of 168 patients had abnormalities in both subsets. The frequency of Th17 cells in the peripheral blood of 79/90 patients (87.8%) was lower than that in healthy controls. In addition, 32.1% of patients had a reduced percentage of natural killer cells, and 32.2% of 149 had impaired T cell proliferation responses to mitogens and/or antigens.

With respect to the B cell compartment, five patients (1.9%) showed increased CD19+ or CD20+ B lymphocytes and 63 (23.9%) showed reduced B cell frequency; only one patient showed increased CD27+CD19+ memory B lymphocyte. Most patients showed normal (*n* = 30, 47.6%) or reduced (*n* = 32, 50.8%) memory B lymphocyte subset.

Low levels of serum IgG, IgA, and IgM (according to age) were observed in 19 (6.5%), 50 (18.3%), and 17 (6.3%) patients with STAT1 GOF mutation, respectively. IgG2 or IgG4 levels were decreased in 3% and 47.6% of 30 patients for whom data were available, respectively, whereas only 1% showed reduced levels of IgG1. Seven patients (2.4%) showed reduced levels of IgG, IgM, and IgA.

**Figure 7:**
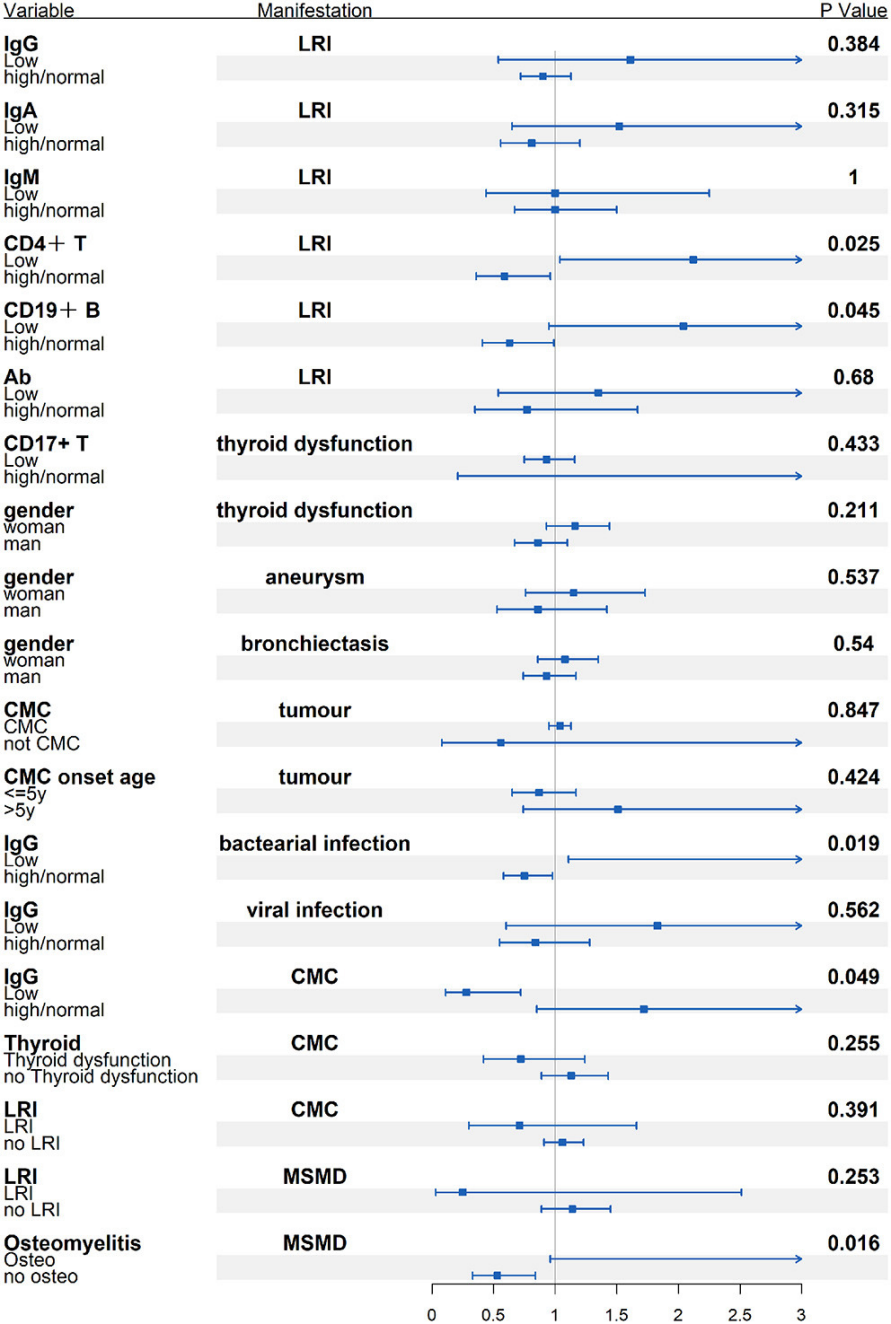
Subgroup analysis of the possible connection between immunological/demographic and clinical manifestations [odds ratio (OR) and 95% confidence intervals (CI)].

There was no difference in serum IgM levels between patients with LRI vs. patients without LRI. STAT1 GOF patients with low IgG levels, low CD4+T lymphocyte numbers, or low CD19+or CD20+B lymphocyte numbers had a higher risk of developing LRI than those without these complications (Figure 7).

### Immunological Investigations of Patients With STAT1 LOF Mutations

Although primary articles documented 39 patients with STAT LOF mutation, different articles reported different data sets. Overall, most patients had normal immune cell populations and immunoglobulin levels. However, 26.7% of 15 patients with STAT1 LOF had high white blood cell counts, 7.7% had high neutrophil counts, 7.1% had high total lymphocyte counts, and 33% had high eosinophil counts (Supplementary Table). None had asthma or eczema in patient with eosinophilia. Analysis of lymphocyte subsets revealed that proliferation of CD4+ T lymphocytes, CD19+ or CD20+ B lymphocytes, and CD27+CD19+ memory B lymphocyte populations was normal. However, 8.3% of 12 patients with STAT1 LOF mutation had high CD8+ T lymphocyte counts, and 20% of five patients with STAT1 LOF mutation had high CD16+CD56+ natural killer cell counts. High levels of serum IgG were observed in 16.7% of 12 patients, whereas 100% had normal IgA, IgM, and IgE levels. Of these, none received IVIG treatment. Only one patient presented with a low titer of antibodies against protein antigens.

### Molecular Findings in Patients With STAT1 GOF Mutations

**Figure 8:**
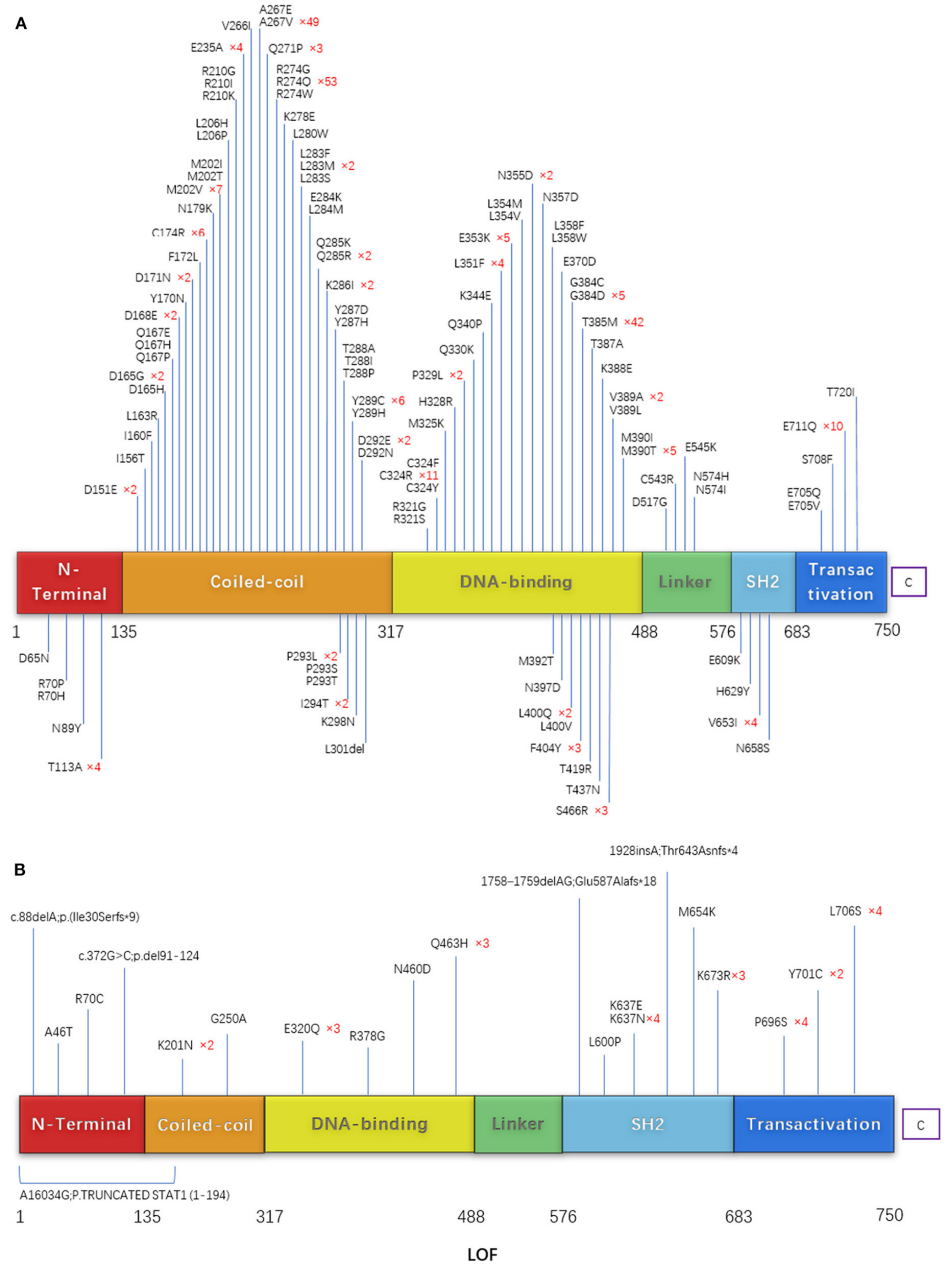
**(A)** Schematic showing the STAT1 protein. **(B)** The locations of mutations that cause STAT1 GOF or LOF mutations are highlighted.

Among the reported STAT1 GOF patients, 111 different mutations were detected. These comprised 110 missense mutations and one deletion; most were in the coiled-coil domain and DNA binding domain (Figure 8A). R274Q was the most common mutation (*n* = 53), followed by A267V (*n* = 49), T385M (*n* = 42), and R274W (*n* = 40). The female/male ratio for these mutations was 0.96, 0.96, 1.10, and 0.62, respectively. Patients with T385M had a higher risk of bronchiectasis than those with the other mutations (*p* < 0.01). The OR (odds ratio) value of T385M/R274Q was 6.19 (95% confidence interval, 2.36–16.25). There was no significant difference between the patients with respect to CMC, bacterial infection, viral infection, thyroid disease, atopy, or tumors (*p* = 0.255, 0.358, 0.371, 0.269, 0.13, and 0.352), respectively. Compared with a previous review (9), more mutations were found with V266I, A267E, L280W, L284M, C324Y, Q340P, L354V, L358F, E609K,E705Q, S708F, the points of T437I,T385K were not included in our study for no concrete data before May 2020.

### Molecular Findings in Patients With STAT1 LOF Mutations

Twenty-one unique mutations in 39 cases with STAT1 LOF have been described. Mutations were located throughout the STAT1 protein: 16 missense mutations, three deletions, one insertion, and one nonsense (affecting more than one exon). Missense mutation at amino acid 637 (K637E, K637N, and K673R) was common (Figure 8B).

## Discussion

Among the reported STAT1 GOF patients, almost all harbored missense mutations located throughout whole exons, mostly in the coiled-coil domain and DNA binding domain. R274Q, A267V, T385M, and R274W were hotspot mutations identified in 41.6% of all patients with STAT1-GOF; therefore, base editing may be a possible choice for further treatment since the 4-editing system could be used for almost half of patients. Patients with T385M had a higher risk of bronchiectasis than those with the other mutations, indicating the need for more aggressive antibiotic treatment or prophylaxis.

Twenty-one unique mutations of STAT1 LOF were located throughout the STAT1 protein; No hotspots were identified. Basically, it is not easy to predict whether any novel missense mutations are a GOF mutation or a LOF mutation; however, frameshift, indel, and nonsense mutations are highly associated with LOF. For alanine substitutions, Kagawa et al. (33) established systemic alanine scanning assay, which correctly predicted 100% of previously reported LOF mutations and 78.1%of known GOF mutations in the CCD/DBD of STAT1. This assay may be a useful tool for evaluating unknown STAT1 variations. But for other sites or more types mutations, more prediction databases remain to be developed.

STAT1 GOF is characterized by a wide variety of manifestations. Mucocutaneous fungal infections by *Candida albicans* were the most common clinical features. LRI and ENT bacterial infections caused by *Staphylococcus aureus, Streptococcus* spp., and *Pseudomonas aeruginosa* were also common in GOF patients, while *Salmonella* infection was common in LOF patients. It is noteworthy that autoimmune disorders seem to be more common in STAT1 GOF than in STAT1 LOF; such conditions involve the endocrine system, Thyroid disease was the most common, indicating that thyroid function and autoantibodies should be examined routinely in patients with STAT1 GOF mutations. In conclusion, CMC alone, CMC with LRI infection, and CMC with autoimmune thyroid disease are clinical hallmarks of patients with STAT1-GOF.

There were fewer patients with STAT1 LOF mutations than with GOF mutations, so we reviewed partial and complete LOF together. In line with previous papers (4, 13–15, 32, 34–36), AR complete STAT1 LOF patients were likely to cause life-threatening infections by mycobacteria and viruses. Patients with AR partial STAT1 LOF mutations were susceptible to infection by intracellular bacteria (Salmonella or mycobacteria) and viruses. Those with AD STAT1 LOF mutations were susceptible to infection by mycobacteria but not by viruses, this is due to varying injury IFN-γ and IFN-α/β responses. Some STAT1 mutants bind STAT2 to form heterodimers and do not impair DNA binding of the complex that maintains ISGF3 activity (36, 37).

Limited data suggest that MSMD with osteomyelitis is a hallmark of STAT1-LOF. Individuals with AR complete LOF had the highest rate of MSMD (90%, compared with AD STAT1 LO, 70.0% and AR partial LOF, 70.0%). Multifocal bone infection might be easily misdiagnosed. The risk of MSMD in STAT1 LOF patients with osteomyelitis was 6.77 times higher than that in those without osteomyelitis (Figure 7). A recent study found that IFN-γ plays a role in inhibiting osteoclastogenesis in human cells, and that this inhibition is weaker in osteoclasts from patients with STAT1 deficiency (38). Another study shows that anti-IFN-γ antibodies induce osteoclast formation, probably mediated by RANKL-induced activation of the NF-κB signaling pathway (39). These findings may partially explain bone destruction in patients with LOF defects in STAT1.

Regarding immunologic manifestations, the majority of patients with GOF had low numbers of Th17 and memory B lymphocytes, and approximately one-third of patients with immunologic record had low numbers of CD4+ T lymphocytes and natural killer cells. Most patients with LOF had normal cell counts although impaired antibody production in response to protein antigens. In general, GOF patients showed more profound immunological abnormality, so Th17 and B cell subset analysis were necessary for differentiation diagnosis of patient with novel heterogeneous missense mutation. Low numbers of CD4+ T or CD19+ CD20+ B cells might be risk factors for LRI (OR = 3.62 and 3.21, respectively). Patients with low IgG levels could be more susceptible to bacterial infection (OR = 4.95). It appears that regular immunoglobulin replacement and prophylactic use of co-azithromycin should be implemented for STAT1 GOF patients, whereas azithromycin or clarithromycin was suggested for STAT1-LOF patients with inflammatory condition and MSMD.

HSCT was performed for 25 patients with STAT1 GOF mutation to treat severe persistent CMC and bacterial infection, systemic viral infections, and severe inflammatory conditions. Forty-one GOF patients died after transplantation, mostly due to severe infections, whereas others died from HLH, multiorgan failure, or interstitial lung disease. The fatal outcomes may be related to the health status of the patients before transplantation or to different preconditioning regimens. Concerning LOF patients, only three underwent HSCT, and two achieved full engraftment and remain alive. HSCT is a curative therapy for STAT1 mutation, but it is currently not the first choice due to the high rate of fatal complications.

Biological therapies such as JAK inhibitors improve symptoms of infection and/or autoimmune disorders in GOF patients. Early studies show that CMC and/or alopecia areata that is resistant to corticosteroids respond well to ruxolitinib (40, 41). Another study reports that the JAK 1/2 inhibitor ruxolitinib not only reverses dysregulated T helper cell responses, but also cures mucocutaneous candidiasis and maintains remission of immune-mediated cytopenias (42). Much higher doses may be needed to normalize chemokine production (43), several reports document increased infections in some patients (42–44); thus, more clinical trials or detailed data are needed for conclusion.

This retrospective review has some limitations. Some information (such as race, nationality, and age of onset) about each manifestation could not be collected. In addition, not all affected mutation carriers underwent functional tests. Patients were treated and evaluated by different clinicians, leading to an incomplete picture of the clinical phenotype. Three articles were excluded due to controversial findings regarding functional changes. Finally, the overall risk may be underestimated because many of the articles were case reports or case series.

## Data Availability Statement

The datasets presented in this study can be found in online repositories. The names of the repository/repositories and accession number(s) can be found in the article/Supplementary Material.

## Author Contributions

YA: concept and protocol design, selection of studies, drafting the review. WZ: principal review author, drafting the protocol, literature search, data extraction and analysis, and drafting the review. XC: participation in the literature search, selection of studies. GG: data checking and analysis. SX: participation in the literature search. LZ: participation in data extraction and analysis. XT: manuscript review and data interpretation. XZ: protocol design, manuscript review, and data interpretation. All authors contributed to the article and approved the submitted version.

## Conflict of Interest

The authors declare that the research was conducted in the absence of any commercial or financial relationships that could be construed as a potential conflict of interest.
