# Supplementary material for: Clinical Relevance of Gain- and Loss-of-Function Germline Mutations in STAT1: A Systematic Review
Source: Front Immunol. 2021 Mar 11;12:654406. doi: 10.3389/fimmu.2021.654406 (PMC7991083; doi:10.3389/fimmu.2021.654406)
Supplement: Supplementary file 1 [file Data_Sheet_1.docx]

**Supplementary table**

**Supplementary Table 1.** Documented pathogens associated with STAT1 GOF mutations

| **Pathogen** | **Results (%)** |
| --- | --- |
| Mucocutaneous fungal infections  Candida albicans  Dematophytes  Fusarium solani  Others  Invasive fungal infections  Candida albicans  Pneumocystis jirovecii  Cryptococcus  Aspergillus  Penicillium marneffei  Others  Bacterial infections  Staphylococcus aureus  Streptococcus spp.  Pseudomonas aeruginosa  Haemophilus influenzae  Klebsiella pneumonia  Salmonella  Clostridium difficile  Others  Mycobacterial infections  M tuberculosis  BCG strain  Others  Viral infections  Herpes simplex  Varicella-zoster  CMV or EBV  Molluscum contagiosum/warts  Encephalitis B virus  JCV  Others | n=244  n=209 (85.7)  n=26 (10.7)  n=1 (0.4)  n=10 (4.1)  n=55  n=10 (18.2)  n=11 (20.0)  n=12 (21.8)  n=11 (20.0)  n=5 (9.1)  n=8 (14.5)  n=138  n=55 (39.9)  n=32 (23.2)  n=22 (15.9)  n=16 (11.6)  n=1 (0.7)  n=1 (0.7)  n=2 (1.4)  n=25 (18.1)  n=40  n=18 (45.0)  n=12 (30.0)  n=13 (32.5)  n=216  n=52 (24.1)  n=73 (33.8)  n=47 (21.8)  n=44 (20.4)  n=1 (0.5)  n=4 (1.9)  n=25 (11.6) |
| Parasitic infections  Toxoplasma gondii  Demodex  Leishmania | n=9  n=1 (11.1)  n=5 (55.6)  n=2 (22.2) |

**Supplementary Table 2.** Documented pathogens associated with STAT1 LOF mutations

| **Pathogens** | **Results (%)** |
| --- | --- |
| Mycobacterial infections  M. tuberculosis  BCG strain  M. avium  M. szulgai  M. kansasii  Not known  Viral infections  Herpes simplex  Varicella-zoster  CMV  EBV  Molluscum contagiosum/warts  HHV  RSV  Adenovirus  Parvovirus  Bacterial infections  Salmonella infection | n=28  n=2 (7.1)  n=17 (60.7)  n=5 (17.9)  n=1 (3.6)  n=1 (3.6)  n=3 (10.7)  n=12  n=5 (41.7)  n=5 (41.7)  n=5 (41.7)  n=6 (50.0)  n=2 (16.7)  n=4 (33.3)  n=4 (33.3)  n=3 (25.0)  n=1 (8.3)  n=5  n=2 (40.0) |
| Shigella  Klebsiella  E. coli  Staphylococcus  Invasive fungal infections  Candida albicans  Aspergillus  Parasitic infections  Toxoplasma gondii (IgG) | n=1 (20.0)  n=1 (20.0)  n=1 (20.0)  n=1 (20.0)  n=2  n=1 (50.0)  n=1 (50.0)  n=1  n=1 (100.0) |

**Supplementary Table 3.** Site of infection in patients with STAT1 GOF mutations

| **Type of infection** | **Patients (%) (n=442)** |
| --- | --- |
| Mucocutaneous fungal infections  Oropharyngeal mycosis  Cutaneous mycosis  Esophageal/genital mycosis  Onychomycosis  Aphtous stomatitis  Scalp mycosis  Invasive fungal infections  Invasive candidiasis  Other invasive infections  Bacterial infections  LRI  ENT  Skin  Others  Mycobacterial infections  Lung disease  Adenitis/skin disease  Disseminated disease  Viral infections  Cutaneous  Systemic | n=363 (82.1)  n=335 (75.8)  n=191 (43.2)  n=186 (42.1)  n=189 (42.8)  n=138 (31.2)  n=56 (12.7)  n=49 (11.1)  n=15 (3.4)  n=36 (8.1)  n=257 (58.1)  n=164 (37.1)  n=150 (33.9)  n=97 (21.9)  n=33 (7.5)  n=41 (9.3)  n=16 (3.6)  n=15 (3.4)  n=13 (2.9)  n=130 (29.4)  n=108 (24.4)  n=35 (7.9) |

**Supplementary Table 4.** Sites of infection in patients with STAT1 LOF mutations

| **Type of infection** | **Results (%)** |
| --- | --- |
| Mycobacterial infections  Lung disease  Adenitis/skin disease  Bone disease  Liver/spleen disease  Brain disease  Ocular disease  Gastrointestinal disease  Disseminated disease  Viral infections  Cutaneous  Brain disease  Lung disease  Liver disease  Systemic  Bacterial infections  LRI  ENT  Skin  Brain  Liver  Bacteremia  Mucocutaneous fungal infections  Invasive fungal infections | n=24  n=4 (16.7)  n=14 (58.3)  n=15 (62.5)  n=4 (16.7)  n=2 (8.3)  n=1 (4.2)  n=1 (4.2)  n=19 (79.2)  n=7  n=4 (57.1)  n=3 (42.9)  n=2 (28.6)  n=1 (14.3)  n=7 (100)  n=5  n=1 (20.0)  n=3 (60.0)  n=1 (20.0)  n=1 (20.0)  n=1 (20.0)  n=3 (60.0)  n=1  n=2 |

**Supplementary Table 5.** Immunological investigation of patients with the STAT1 GOF mutation

| **Biological investigation** | **Patients (n)** | **Normal (%)** | **Low (%)** | **High (%)** |
| --- | --- | --- | --- | --- |
| Total lymphocytes | 291 | 75.6 | 23.0 | 1.4 |
| CD4+ T lymphocytes | 276 | 68.1 | 30.8 | 1.1 |
| CD8+ T lymphocytes | 268 | 79.5 | 17.9 | 2.6 |
| CD19+ or CD20+ B lymphocytes | 264 | 74.2 | 23.9 | 1.9 |
| CD27+CD19+ memory B lymphocytes | 63 | 47.6 | 50.8 | 1.6 |
| CD16+CD56+ natural killer cells | 209 | 66.5 | 32.1 | 1.4 |
| CD3+IL-17+ or CD4+IL-17+ T lymphocytes | 90 | 12.2 | 87.8 | 0 |
| T cell proliferation (mitogen and/or antigen) | 149 | 67.8 | 32.2 | 0 |
| IgG levels (g/L) | 294 | 73.1 | 6.5 | 20.4 |
| IgA levels (g/L) | 273 | 75.8 | 18.3 | 6.2 |
| IgM levels (g/L) | 272 | 93.0 | 6.3 | 0.7 |
| IgG1 levels (g/L) | 102 | 99.0 | 1.0 | 0 |
| IgG2 levels (g/L) | 107 | 60.7 | 39.3 | 0 |
| IgG4 levels (g/L) | 105 | 52.4 | 47.6 | 0 |
| IgE levels (g/L) | 162 | 94.4 | 1.2 | 4.3 |
| Antibodies against protein antigens | 134 | 72.4 | 27.6 | 0 |

**Supplementary Table 6.** Immunological investigation of patients with the STAT1 LOF mutation

| **Biological investigation** | **Patients (n)** | **Normal (%)** | **Low (%)** | **High (%)** |
| --- | --- | --- | --- | --- |
| WBC | 15 | 73.3 | 0.0 | 26.7 |
| Neutrophils | 13 | 92.3 | 0.0 | 7.7 |
| Monocyte | 4 | 100.0 | 0.0 | 0.0 |
| Eosinophils | 6 | 66.7 | 0.0 | 33.3 |
| Total lymphocyte | 14 | 92.9 | 0.0 | 7.1 |
| CD4+ T lymphocytes | 12 | 100.0 | 0.0 | 0.0 |
| CD8+ T lymphocytes | 12 | 91.7 | 0.0 | 8.3 |
| CD19+ or CD20+ B lymphocytes | 12 | 100.0 | 0.0 | 0.0 |
| CD27+CD19+ memory B lymphocytes | 2 | 100.0 | 0.0 | 0.0 |
| CD16+CD56+ natural killer cells | 5 | 80.0 | 0.0 | 20.0 |
| T cell proliferation (mitogen and/or antigen) | 3 | 100.0 | 0.0 | 0.0 |
| IgG levels (g/L) | 12 | 83.3 | 0.0 | 16.7 |
| IgA levels (g/L) | 10 | 100.0 | 0.0 | 0.0 |
| IgM levels (g/L) | 10 | 100.0 | 0.0 | 0.0 |
| IgE levels (g/L) | 10 | 100.0 | 0.0 | 0.0 |
| Antibodies against protein antigens | 4 | 75.0 | 25.0 | 0.0 |

WBC, White blood cell.

**Supplementary Figure**

**Supplementary Figure 1.** Kaplan–Meier curves. (A) Onset of CMC for patients with GOF. (B) Overall survival curves for patients with GOF. (C) Overall survival curves for patients with LOF.


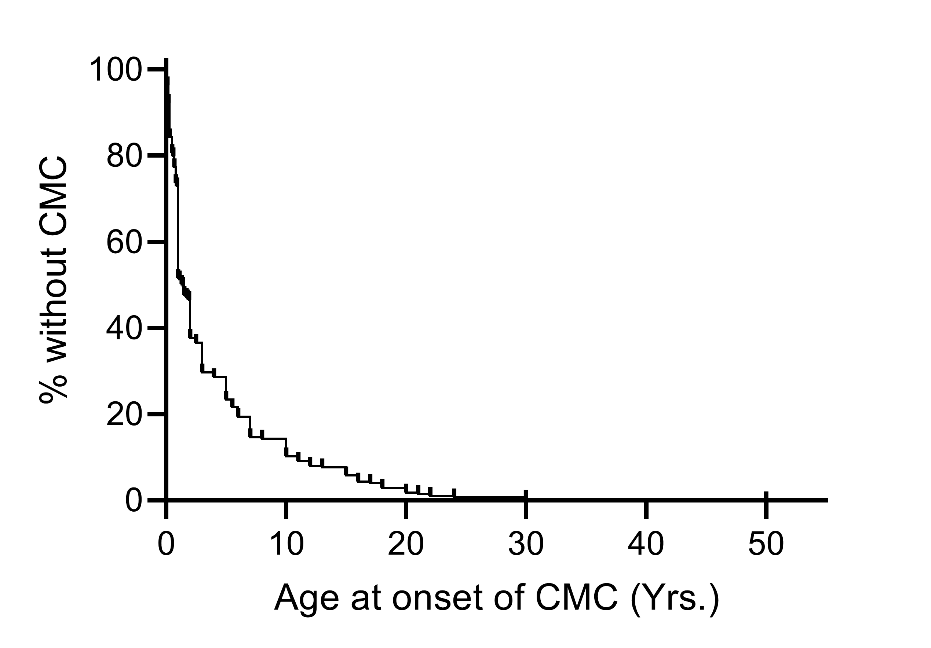


Figure 1A.


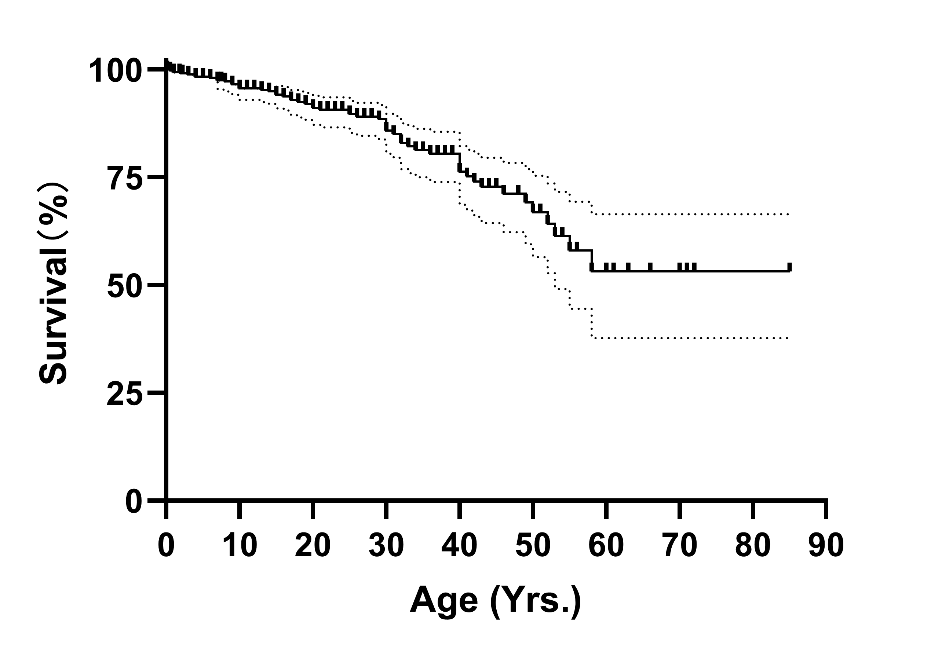


Figure 1B.


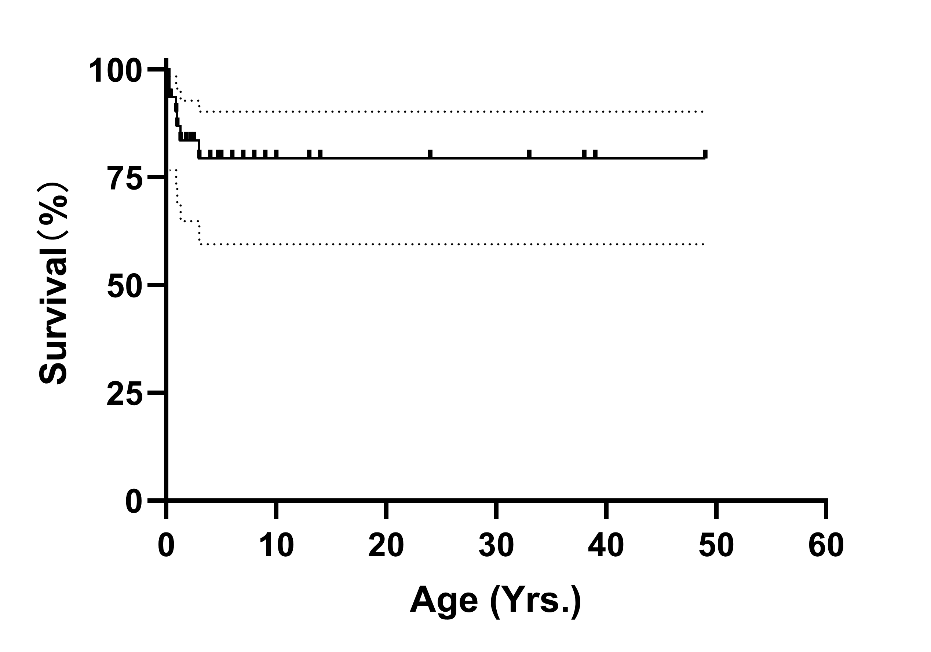


Figure 1C.

**Supplementary Figure 2.** Several strains of mycobacteria.


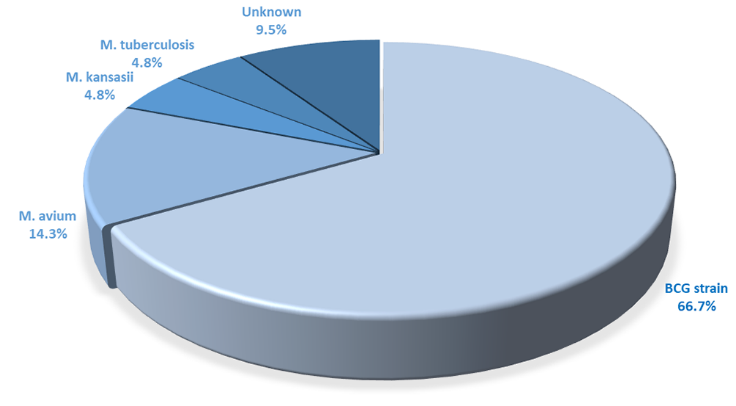


Articles included in this review (n=132):

1. Corbeel L, Ceuppens JL, Van den Berghe G, Claeys H, Casteels-Van Daele M. Immunological observations before and after successful treatment of chronic mucocutaneous candidiasis with ketoconazole and transfer factor. *Eur J Pediatr* (1984) 143(1):45-8. Epub 1984/11/01. doi: 10.1007/bf00442747. PubMed PMID: 6096150.

2. Nagashima T, Miyanoshita A, Sakiyama Y, Ozaki Y, Stan AC, Nagashima K. Cerebral vasculitis in chronic mucocutaneous candidiasis: autopsy case report. *Neuropathology : official journal of the Japanese Society of Neuropathology* (2000) 20(4):309-14. Epub 2001/02/24. doi: 10.1046/j.1440-1789.2000.00352.x. PubMed PMID: 11211056.

3. Atkinson TP, Schaffer AA, Grimbacher B, Schroeder HW, Jr., Woellner C, Zerbe CS, et al. An immune defect causing dominant chronic mucocutaneous candidiasis and thyroid disease maps to chromosome 2p in a single family. *Am J Hum Genet* (2001) 69(4):791-803. Epub 2001/08/23. doi: 10.1086/323611. PubMed PMID: 11517424; PubMed Central PMCID: PMCPMC1226065.

4. Myhre AG, Stray-Pedersen A, Spangen S, Eide E, Veimo D, Knappskog PM, et al. Chronic mucocutaneous candidiasis and primary hypothyroidism in two families. *Eur J Pediatr* (2004) 163(10):604-11. Epub 2004/08/04. doi: 10.1007/s00431-004-1516-8. PubMed PMID: 15290270.

5. Koch D, Lilic D, Carmichael AJ. Autosomal dominant chronic mucocutaneous candidiasis and primary hypothyroidism complicated by oesophageal carcinoma. *Clinical and experimental dermatology* (2009) 34(8):e818-20. Epub 2009/09/26. doi: 10.1111/j.1365-2230.2009.03561.x. PubMed PMID: 19778308.

6. Firinu D, Massidda O, Lorrai MM, Serusi L, Peralta M, Barca MP, et al. Successful treatment of chronic mucocutaneous candidiasis caused by azole-resistant Candida albicans with posaconazole. *Clinical & developmental immunology* (2011) 2011:283239. Epub 2011/01/05. doi: 10.1155/2011/283239. PubMed PMID: 21197459; PubMed Central PMCID: PMCPMC3003968.

7. Liu L, Okada S, Kong XF, Kreins AY, Cypowyj S, Abhyankar A, et al. Gain-of-function human STAT1 mutations impair IL-17 immunity and underlie chronic mucocutaneous candidiasis. *Journal of Experimental Medicine* (2011) 208(18):1635-48. doi: 10.1084/jem.20110958.

8. Smeekens SP, Plantinga TS, van de Veerdonk FL, Heinhuis B, Hoischen A, Joosten LA, et al. STAT1 hyperphosphorylation and defective IL12R/IL23R signaling underlie defective immunity in autosomal dominant chronic mucocutaneous candidiasis. *PLoS One* (2011) 6(12):e29248. Epub 2011/12/24. doi: 10.1371/journal.pone.0029248. PubMed PMID: 22195034; PubMed Central PMCID: PMCPMC3237610.

9. van de Veerdonk FL, Plantinga TS, Hoischen A, Smeekens SP, Joosten LAB, Gilissen C, et al. STAT1 Mutations in Autosomal Dominant Chronic Mucocutaneous Candidiasis. *New England Journal of Medicine* (2011) 365(1):54-61. doi: 10.1056/NEJMoa1100102. PubMed PMID: WOS:000292459400009.

10. Bader O, Weig MS, Gross U, Schon MP, Mempel M, Buhl T. Photo quiz. A 32-year-old man with ulcerative mucositis, skin lesions, and nail dystrophy. Chronic mucocutaneous candidiasis by multidrug-resistant Candida albicans. *Clinical infectious diseases : an official publication of the Infectious Diseases Society of America* (2012) 54(7):972, 1035-6. Epub 2012/03/10. doi: 10.1093/cid/cir958. PubMed PMID: 22403094.

11. Hori T, Ohnishi H, Teramoto T, Tsubouchi K, Naiki T, Hirose Y, et al. Autosomal-dominant chronic mucocutaneous candidiasis with stat1-mutation can be complicated with chronic active hepatitis and hypothyroidism. *Journal of Clinical Immunology* (2012) 32(6):1213-20. doi: 10.1007/s10875-012-9744-6.

12. Lee PP, Chan KW, Lee TL, Ho MH, Chen XY, Li CH, et al. Penicilliosis in children without HIV infection--are they immunodeficient? *Clinical infectious diseases : an official publication of the Infectious Diseases Society of America* (2012) 54(2):e8-e19. Epub 2011/11/09. doi: 10.1093/cid/cir754. PubMed PMID: 22065867.

13. Takezaki S, Yamada M, Kato M, Park M-j, Maruyama K, Yamazaki Y, et al. Chronic Mucocutaneous Candidiasis Caused by a Gain-of-Function Mutation in the STAT1 DNA-Binding Domain. *Journal of Immunology* (2012) 189(3):1521-6. doi: 10.4049/jimmunol.1200926. PubMed PMID: WOS:000306599100049.

14. Toth B, Mehes L, Tasko S, Szalai Z, Tulassay Z, Cypowyj S, et al. Herpes in STAT1 gain-of-function mutation [corrected]. *Lancet* (2012) 379(9835):2500. Epub 2012/07/04. doi: 10.1016/s0140-6736(12)60365-1. PubMed PMID: 22748593.

15. Yamazaki Y, Yamada M, Takezaki S, Kato M, Park MJ, Maruyama K, et al. Chronic mucocutaneous candidiasis caused by a gain-offunction mutation in the STAT1 dnabinding domain. *Journal of Clinical Immunology* (2012) 32:S100.

16. Erratum: Gain-of-function STAT1 mutations are associated with PD-L1 overexpression and a defect in B-cell survival (Computer Graphics Forum (2013) 131 (1691-3)). *Journal of Allergy and Clinical Immunology* (2013) 132(6):1460. doi: 10.1016/j.jaci.2013.10.001).

17. Al Rushood M, McCusker C, Mazer B, Alizadehfar R, Grimbacher B, Depner M, et al. Autosomal dominant cases of chronic mucocutaneous candidiasis segregates with mutations of signal transducer and activator of transcription 1, but not of toll-like receptor 3. *Journal of Pediatrics* (2013) 163(1):277-9. doi: 10.1016/j.jpeds.2013.02.040.

18. Aldave JC, Cachay E, Nunez L, Chunga A, Murillo S, Cypowyj S, et al. A 1-year-old girl with a gain-of-function STAT1 mutation treated with hematopoietic stem cell transplantation. *J Clin Immunol* (2013) 33(8):1273-5. Epub 2013/10/10. doi: 10.1007/s10875-013-9947-5. PubMed PMID: 24105462.

19. Romberg N, Morbach H, Lawrence MG, Kim S, Kang I, Holland SM, et al. Gain-of-function STAT1 mutations are associated with PD-L1 overexpression and a defect in B-cell survival. *Journal of Allergy and Clinical Immunology* (2013) 131(6):1691-3. doi: 10.1016/j.jaci.2013.01.004.

20. Sampaio EP, Hsu AP, Pechacek J, Bax HI, Dias DL, Paulson ML, et al. Signal transducer and activator of transcription 1 (STAT1) gain-of-function mutations and disseminated coccidioidomycosis and histoplasmosis. *Journal of Allergy and Clinical Immunology* (2013) 131(6):1624-34.e17. doi: 10.1016/j.jaci.2013.01.052.

21. Soltesz B, Toth B, Shabashova N, Bondarenko A, Okada S, Cypowyj S, et al. New and recurrent gain-of-function STAT1 mutations in patients with chronic mucocutaneous candidiasis from Eastern and Central Europe. *J Med Genet* (2013) 50(9):567-78. Epub 2013/05/28. doi: 10.1136/jmedgenet-2013-101570. PubMed PMID: 23709754; PubMed Central PMCID: PMCPMC3756505.

22. Uzel G, Sampaio EP, Lawrence MG, Hsu AP, Hackett M, Dorsey MJ, et al. Dominant gain-of-function STAT1 mutations in FOXP3 wild-type immune dysregulation-polyendocrinopathy-enteropathy-X-linked-like syndrome. *Journal of Allergy and Clinical Immunology* (2013) 131(6):1611-23.e3. doi: 10.1016/j.jaci.2012.11.054.

23. Wang X, Lin Z, Gao L, Wang A, Wan Z, Chen W, et al. Exome sequencing reveals a signal transducer and activator of transcription 1 (STAT1) mutation in a child with recalcitrant cutaneous fusariosis. *J Allergy Clin Immunol* (2013) 131(4):1242-3. Epub 2012/12/19. doi: 10.1016/j.jaci.2012.11.005. PubMed PMID: 23245795.

24. Frans G, Moens L, Schaballie H, Van Eyck L, Borgers H, Wuyts M, et al. Gain-of-function mutations in signal transducer and activator of transcription 1 (STAT1): Chronic mucocutaneous candidiasis accompanied by enamel defects and delayed dental shedding. *Journal of Allergy and Clinical Immunology* (2014) 134(5):1209-13. doi: 10.1016/j.jaci.2014.05.044. PubMed PMID: WOS:000344938900034.

25. Kumar N, Hanks ME, Chandrasekaran P, Davis BC, Hsu AP, Van Wagoner NJ, et al. Gain-of-function signal transducer and activator of transcription 1 (STAT1) mutation-related primary immunodeficiency is associated with disseminated mucormycosis. *Journal of Allergy and Clinical Immunology* (2014) 134(1):236-9. doi: 10.1016/j.jaci.2014.02.037.

26. Lee PPW, Mao H, Yang W, Chan KW, Ho MHK, Lee TL, et al. Penicillium marneffei infection and impaired IFN-γ immunity in humans with autosomal-dominant gain-of-phosphorylation STAT1 mutations. *Journal of Allergy and Clinical Immunology* (2014) 133(3):894-6.e5. doi: 10.1016/j.jaci.2013.08.051.

27. Mekki N, Ben-Mustapha I, Liu L, Boussofara L, Okada S, Cypowyj S, et al. IL-17 T cells' defective differentiation in vitro despite normal range ex vivo in chronic mucocutaneous candidiasis due to STAT1 mutation. *Journal of Investigative Dermatology* (2014) 134(4):1155-7. doi: 10.1038/jid.2013.480.

28. Mizoguchi Y, Tsumura M, Okada S, Hirata O, Minegishi S, Imai K, et al. Simple diagnosis of STAT1 gain-of-function alleles in patients with chronic mucocutaneous candidiasis. *Journal of Leukocyte Biology* (2014) 95(4):667-76. doi: 10.1189/jlb.0513250.

29. Yamazaki Y, Yamada M, Kawai T, Morio T, Onodera M, Ueki M, et al. Two novel gain-of-function mutations of stat1 responsible for chronic mucocutaneous candidiasis disease: Impaired production of il-17a and il-22, and the presence of anti-il-17f autoantibody. *Journal of Immunology* (2014) 193(10):4880-7. doi: 10.4049/jimmunol.1401467.

30. Higgins E, Al Shehri T, McAleer MA, Conlon N, Feighery C, Lilic D, et al. Use of ruxolitinib to successfully treat chronic mucocutaneous candidiasis caused by gain-of-function signal transducer and activator of transcription 1 (STAT1) mutation. *Journal of Allergy and Clinical Immunology* (2015) 135(2):551-3. doi: 10.1016/j.jaci.2014.12.1867.

31. Kilic SS, Puel A, Casanova JL. Orf Infection in a Patient with Stat1 Gain-of-Function. *Journal of Clinical Immunology* (2015) 35(1):80-3. doi: 10.1007/s10875-014-0111-7.

32. Martinez-Martinez L, Martinez-Saavedra MT, Fuentes-Prior P, Barnadas M, Rubiales MV, Noda J, et al. A novel gain-of-function STAT1 mutation resulting in basal phosphorylation of STAT1 and increased distal IFN-gamma-mediated responses in chronic mucocutaneous candidiasis. *Mol Immunol* (2015) 68(2 Pt C):597-605. Epub 2015/10/31. doi: 10.1016/j.molimm.2015.09.014. PubMed PMID: 26514428.

33. Nielsen J, Kofod-Olsen E, Spaun E, Larsen CS, Christiansen M, Mogensen TH. A STAT1-gain-of-function mutation causing Th17 deficiency with chronic mucocutaneous candidiasis, psoriasiform hyperkeratosis and dermatophytosis. *BMJ Case Reports* (2015) 2015. doi: 10.1136/bcr-2015-211372.

34. Tanimura M, Dohi K, Hirayama M, Sato Y, Sugiura E, Nakajima H, et al. Recurrent inflammatory aortic aneurysms in chronic mucocutaneous candidiasis with a gain-of-function STAT1 mutation. *International Journal of Cardiology* (2015) 196:88-90. doi: 10.1016/j.ijcard.2015.05.183.

35. van de Veerdonk FL, Koenen HJPM, van der Velden WJFM, van der Meer JWM, Netea MG. Immunotherapy with G-CSF in patients with chronic mucocutaneous. *Immunology Letters* (2015) 167(1):54-6. doi: 10.1016/j.imlet.2015.05.008. PubMed PMID: WOS:000360596200009.

36. Baris S, Alroqi F, Kiykim A, Karakoc-Aydiner E, Ogulur I, Ozen A, et al. Severe Early-Onset Combined Immunodeficiency due to Heterozygous Gain-of-Function Mutations in STAT1. *J Clin Immunol* (2016) 36(7):641-8. Epub 2016/07/06. doi: 10.1007/s10875-016-0312-3. PubMed PMID: 27379765; PubMed Central PMCID: PMCPMC5556363.

37. Chang MT, Schwam ZG, Hajek MA, Paskhover B, Judson BL. Severe epistaxis due to aberrant vasculature in a patient with STAT-1 mutation. *Head and Neck* (2016) 38(3):E68-E70. doi: 10.1002/hed.24165.

38. Depner M, Fuchs S, Raabe J, Frede N, Glocker C, Doffinger R, et al. The Extended Clinical Phenotype of 26 Patients with Chronic Mucocutaneous Candidiasis due to Gain-of-Function Mutations in STAT1. *J Clin Immunol* (2016) 36(1):73-84. Epub 2015/11/26. doi: 10.1007/s10875-015-0214-9. PubMed PMID: 26604104; PubMed Central PMCID: PMCPMC4718942.

39. Dhalla F, Fox H, Davenport EE, Sadler R, Anzilotti C, van Schouwenburg PA, et al. Chronic mucocutaneous candidiasis: characterization of a family with STAT-1 gain-of-function and development of an ex-vivo assay for Th17 deficiency of diagnostic utility. *Clinical and Experimental Immunology* (2016) 184(2):216-27. doi: 10.1111/cei.12746.

40. Dotta L, Scomodon O, Padoan R, Timpano S, Plebani A, Soresina A, et al. Clinical heterogeneity of dominant chronic mucocutaneous candidiasis disease: Presenting as treatment-resistant candidiasis and chronic lung disease. *Clinical Immunology* (2016) 164:1-9. doi: 10.1016/j.clim.2015.12.010.

41. Dotta L, Scomodon O, Padoan R, Timpano S, Plebani A, Soresina A, et al. Clinical and immunological data of nine patients with chronic mucocutaneous candidiasis disease. *Data Brief* (2016) 7:311-5. doi: 10.1016/j.dib.2016.02.040.

42. Giardino G, Somma D, Cirillo E, Ruggiero G, Terrazzano G, Rubino V, et al. Novel STAT1 gain-of-function mutation and suppurative infections. *Pediatric Allergy and Immunology* (2016) 27(2):220-3. doi: 10.1111/pai.12496.

43. Kataoka S, Muramatsu H, Okuno Y, Hayashi Y, Mizoguchi Y, Tsumura M, et al. Extrapulmonary tuberculosis mimicking Mendelian susceptibility to mycobacterial disease in a patient with signal transducer and activator of transcription 1 (STAT1) gain-of-function mutation. *Journal of Allergy and Clinical Immunology* (2016) 137(2):619-22. doi: 10.1016/j.jaci.2015.06.028.

44. Kobbe R, Kolster M, Fuchs S, Schulze-Sturm U, Jenderny J, Kochhan L, et al. Common variable immunodeficiency, impaired neurological development and reduced numbers of T regulatory cells in a 10-year-old boy with a STAT1 gain-of-function mutation. *Gene* (2016) 586(2):234-8. doi: 10.1016/j.gene.2016.04.006.

45. Liu G, Li C, Yang J, Zhang Y, Luo Y, Xia Y, et al. A case of chronic mucocutaneous candidiasis caused by STAT1 gain-of-function mutation and literature review. *Chinese Journal of Applied Clinical Pediatrics* (2016) 31(18):1422-5. PubMed PMID: CSCD:5798167.

46. Mossner R, Diering N, Bader O, Forkel S, Overbeck T, Gross U, et al. Ruxolitinib Induces Interleukin 17 and Ameliorates Chronic Mucocutaneous Candidiasis Caused by STAT1 Gain-of-Function Mutation. *Clinical infectious diseases : an official publication of the Infectious Diseases Society of America* (2016) 62(7):951-3. Epub 2016/01/21. doi: 10.1093/cid/ciw020. PubMed PMID: 26787170.

47. Sobh A, Chou J, Schneider L, Geha RS, Massaad MJ. Chronic mucocutaneous candidiasis associated with an SH2 domain gain-of-function mutation that enhances STAT1 phosphorylation. *Journal of Allergy and Clinical Immunology* (2016) 138(1):297-9. doi: 10.1016/j.jaci.2015.12.1320.

48. Toubiana J, Okada S, Hiller J, Oleastro M, Gomez ML, Becerra JCA, et al. Heterozygous STAT1 gain-of-function mutations underlie an unexpectedly broad clinical phenotype. *Blood* (2016) 127(25):3154-64. doi: 10.1182/blood-2015-11-679902.

49. Wessell KR, Tcheurekdjian H, Hostoffer R. Autosomal dominant transmission of signal transduction and activator of transcription 1 (STAT1) mutation (Thr385Met) and extended lifespan. *LymphoSign Journal* (2016) 3(1):13-7. doi: 10.14785/lpsn-2015-0013.

50. Zerbe CS, Marciano BE, Katial RK, Santos CB, Adamo N, Hsu AP, et al. Progressive Multifocal Leukoencephalopathy in Primary Immune Deficiencies: Stat1 Gain of Function and Review of the Literature. *Clinical Infectious Diseases* (2016) 62(8):986-94. doi: 10.1093/cid/civ1220.

51. Becerra JCA, Rojas EC. A 3-year-old girl with recurrent infections and autoimmunity due to a STAT1 gain-of-function mutation: The expanding clinical presentation of primary immunodeficiencies. *Frontiers in Pediatrics* (2017) 5. doi: 10.3389/fped.2017.00055.

52. Boudjemaa S, Dainese L, Heritier S, Masserot C, Hachemane S, Casanova JL, et al. Disseminated Bacillus Calmette-Guerin Osteomyelitis in Twin Sisters Related to STAT1 Gene Deficiency. *Pediatric and developmental pathology : the official journal of the Society for Pediatric Pathology and the Paediatric Pathology Society* (2017) 20(3):255-61. Epub 2017/05/20. doi: 10.1177/1093526616686255. PubMed PMID: 28521627.

53. Breuer O, Daum H, Cohen-Cymberknoh M, Unger S, Shoseyov D, Stepensky P, et al. Autosomal dominant gain of function STAT1 mutation and severe bronchiectasis. *Respiratory Medicine* (2017) 126:39-45. doi: 10.1016/j.rmed.2017.03.018.

54. Dadak M, Jacobs R, Skuljec J, Jirmo AC, Yildiz Ö, Donnerstag F, et al. Gain-of-function STAT1 mutations are associated with intracranial aneurysms. *Clinical Immunology* (2017) 178:79-85. doi: 10.1016/j.clim.2017.01.012.

55. Eren Akarcan S, Ulusoy Severcan E, Edeer Karaca N, Isik E, Aksu G, Migaud M, et al. Gain-of-Function Mutations in STAT1: A Recently Defined Cause for Chronic Mucocutaneous Candidiasis Disease Mimicking Combined Immunodeficiencies. *Case Reports Immunol* (2017) 2017:2846928. Epub 2017/12/21. doi: 10.1155/2017/2846928. PubMed PMID: 29259832; PubMed Central PMCID: PMCPMC5702932.

56. Eslami N, Tavakol M, Mesdaghi M, Gharegozlou M, Casanova JL, Puel A, et al. A Gain-of-function mutation of stat1: A novel genetic factor contributing to chronic mucocutaneous candidiasis. *Acta Microbiologica et Immunologica Hungarica* (2017) 64(2):191-201. doi: 10.1556/030.64.2017.014.

57. Koo S, Kejariwal D, Al-Shehri T, Dhar A, Lilic D. Oesophageal candidiasis and squamous cell cancer in patients with gain-of-function STAT1 gene mutation. *United European Gastroenterology Journal* (2017) 5(5):625-31. doi: 10.1177/2050640616684404.

58. Lee WI, Chen CC, Jaing TH, Ou LS, Hsueh C, Huang JL. A Nationwide Study of Severe and Protracted Diarrhoea in Patients with Primary Immunodeficiency Diseases. *Scientific reports* (2017) 7(1):3669. doi: 10.1038/s41598-017-03967-4.

59. Meesilpavikkai K, Dik WA, Schrijver B, Nagtzaam NM, van Rijswijk A, Driessen GJ, et al. A Novel Heterozygous Mutation in the STAT1 SH2 Domain Causes Chronic Mucocutaneous Candidiasis, Atypically Diverse Infections, Autoimmunity, and Impaired Cytokine Regulation. *Front Immunol* (2017) 8:274. Epub 2017/03/30. doi: 10.3389/fimmu.2017.00274. PubMed PMID: 28348565; PubMed Central PMCID: PMCPMC5346540.

60. Pedraza-Sanchez S, Lezana-Fernandez JL, Gonzalez Y, Martinez-Robles L, Ventura-Ayala ML, Sadowinski-Pine S, et al. Disseminated Tuberculosis and Chronic Mucocutaneous Candidiasis in a Patient with a Gain-of-Function Mutation in Signal Transduction and Activator of Transcription 1. *Front Immunol* (2017) 8:1651. Epub 2017/12/23. doi: 10.3389/fimmu.2017.01651. PubMed PMID: 29270166; PubMed Central PMCID: PMCPMC5723642.

61. Peter J, Onyango VC. When you cannot beat back the mould: Chronic mucocutaneous candidiasis. *Current Allergy and Clinical Immunology* (2017) 30(2):116-21.

62. Rae W, Ward D, Mattocks CJ, Gao Y, Pengelly RJ, Pate SV, et al. Autoimmunity/inflammation in a monogenic primary immunodeficiency cohort. *Clinical and Translational Immunology* (2017) 6(9). doi: 10.1038/cti.2017.38.

63. Second J, Korganow AS, Jannier S, Puel A, Lipsker D. Rosacea and demodicidosis associated with gain-of-function mutation in STAT1. *Journal of the European Academy of Dermatology and Venereology* (2017) 31(12):e542-e4. doi: 10.1111/jdv.14413.

64. Tabellini G, Vairo D, Scomodon O, Tamassia N, Ferraro RM, Patrizi O, et al. Impaired natural killer cell functions in patients with signal transducer and activator of transcription 1 (STAT1) gain-of-function mutations. *Journal of Allergy and Clinical Immunology* (2017) 140(2):553-64.e4. doi: 10.1016/j.jaci.2016.10.051.

65. Weinacht KG, Charbonnier L-M, Alroqi F, Plant A, Qiao Q, Wu H, et al. Ruxolitinib reverses dysregulated T helper cell responses and controls autoimmunity caused by a novel signal transducer and activator of transcription 1 (STAT1) gain-of-function mutation. *Journal of Allergy and Clinical Immunology* (2017) 139(5):1629-+. doi: 10.1016/j.jaci.2016.11.022. PubMed PMID: WOS:000400465300024.

66. Zimmerman O, Rosler B, Zerbe CS, Rosen LB, Hsu AP, Uzel G, et al. Risks of Ruxolitinib in STAT1 Gain-of-Function-Associated Severe Fungal Disease. *Open Forum Infect Dis* (2017) 4(4):ofx202. Epub 2017/12/12. doi: 10.1093/ofid/ofx202. PubMed PMID: 29226168; PubMed Central PMCID: PMCPMC5714179.

67. Al Dhanhani H, Al Shehri T, Lilic D, Buddles M, Kisand K, Maccari ME, et al. Double Trouble? CMC with a Mutation in both AIRE and STAT1. *Journal of Clinical Immunology* (2018) 38(6):635-7. doi: 10.1007/s10875-018-0536-5.

68. Bernasconi AR, Yancoski J, Villa M, Oleastro MM, Galicchio M, Rossi JG. Increased STAT1 Amounts Correlate with the Phospho-STAT1 Level in STAT1 Gain-of-function Defects. *Journal of Clinical Immunology* (2018) 38(7):745-7. doi: 10.1007/s10875-018-0557-0.

69. Bloomfield M, Kanderová V, Paračková Z, Vrabcová P, Svatoň M, Froňková E, et al. Utility of ruxolitinib in a child with chronic mucocutaneous candidiasis caused by a novel STAT1 gain-of-function mutation. *Journal of Clinical Immunology* (2018) 38(5):589-601. doi: 10.1007/s10875-018-0519-6.

70. Forbes LR, Vogel TP, Cooper MA, Castro-Wagner J, Schussler E, Weinacht KG, et al. Jakinibs for the treatment of immune dysregulation in patients with gain-of-function signal transducer and activator of transcription 1 (STAT1) or STAT3 mutations. *Journal of Allergy and Clinical Immunology* (2018) 142(5):1665-9. doi: 10.1016/j.jaci.2018.07.020.

71. Hartono SP, Vargas-Hernández A, Ponsford MJ, Chinn IK, Jolles S, Wilson K, et al. Novel STAT1 Gain-of-Function Mutation Presenting as Combined Immunodeficiency. *Journal of Clinical Immunology* (2018) 38(7):753-6. doi: 10.1007/s10875-018-0554-3.

72. Huh HJ, Jhun BW, Choi SR, Kim YJ, Yun SA, Nham E, et al. Bronchiectasis and recurrent respiratory infections with a de novo STAT1 gain-of-function variant: First case in Korea. *Yonsei Medical Journal* (2018) 59(8):1004-7. doi: 10.3349/ymj.2018.59.8.1004.

73. Leiding JW, Okada S, Hagin D, Abinun M, Shcherbina A, Balashov DN, et al. Hematopoietic stem cell transplantation in patients with gain-of-function signal transducer and activator of transcription 1 mutations. *Journal of Allergy and Clinical Immunology* (2018) 141(2):704-17.e5. doi: 10.1016/j.jaci.2017.03.049.

74. Li PH, Lee PPW, Fung SL, Lau CS, Lau YL. Chronic mucocutaneous candidiasis-more than just skin deep. *Hong Kong Medical Journal* (2018) 24(4):423-5. doi: 10.12809/hkmj166048. PubMed PMID: WOS:000442116900015.

75. Luis BAL, Calva-Mercado JJ. Recurrent Spontaneous Intestinal Perforation due to STAT1 GOF Mutation. *The American journal of gastroenterology* (2018) 113(7):1057-8. Epub 2018/06/09. doi: 10.1038/s41395-018-0089-5. PubMed PMID: 29880965.

76. Meesilpavikkai K, Dik WA, Schrijver B, Nagtzaam NMA, Posthumus-van Sluijs SJ, van Hagen PM, et al. Baricitinib treatment in a patient with a gain-of-function mutation in signal transducer and activator of transcription 1 (STAT1). *J Allergy Clin Immunol* (2018) 142(1):328-30.e2. Epub 2018/05/08. doi: 10.1016/j.jaci.2018.02.045. PubMed PMID: 29729898.

77. Ovadia A, Sharfe N, Hawkins C, Laughlin S, Roifman CM. Two different STAT1 gain-of-function mutations lead to diverse IFN-γ-mediated gene expression. *npj Genomic Medicine* (2018) 3(1). doi: 10.1038/s41525-018-0063-6.

78. Pedraza-Sanchez S, Mendez-Leon JI, Gonzalez Y, Ventura-Ayala ML, Herrera MT, Lezana-Fernandez JL, et al. Oral Administration of Human Polyvalent IgG by Mouthwash as an Adjunctive Treatment of Chronic Oral Candidiasis. *Front Immunol* (2018) 9:2956. Epub 2019/01/11. doi: 10.3389/fimmu.2018.02956. PubMed PMID: 30627128; PubMed Central PMCID: PMCPMC6309162.

79. Ro T, Sood A, Kelly KJ, Morrell DS. What Is the Cause of the Chronic Erythematous Scaling Plaques on This 22-Month-Old Girl and Her Family? *Clinical Pediatrics* (2018) 57(7):874-8. doi: 10.1177/0009922817738347.

80. Sampaio EP, Ding L, Rose SR, Cruz P, Hsu AP, Kashyap A, et al. Novel signal transducer and activator of transcription 1 mutation disrupts small ubiquitin-related modifier conjugation causing gain of function. *Journal of Allergy and Clinical Immunology* (2018) 141(5):1844-53.e2. doi: 10.1016/j.jaci.2017.07.027.

81. Smyth AE, Kaleviste E, Snow A, Kisand K, McMahon CJ, Cant AJ, et al. Aortic Calcification in a Patient with a Gain-of-Function STAT1 Mutation. *Journal of Clinical Immunology* (2018) 38(4):468-70. doi: 10.1007/s10875-018-0513-z.

82. Vargas-Hernández A, Mace EM, Zimmerman O, Zerbe CS, Freeman AF, Rosenzweig S, et al. Ruxolitinib partially reverses functional natural killer cell deficiency in patients with signal transducer and activator of transcription 1 (STAT1) gain-of-function mutations. *Journal of Allergy and Clinical Immunology* (2018) 141(6):2142-55.e5. doi: 10.1016/j.jaci.2017.08.040.

83. Al Shehri T, Gilmour K, Gothe F, Loughlin S, Bibi S, Rowan AD, et al. Novel Gain-of-Function Mutation in Stat1 Sumoylation Site Leads to CMC/CID Phenotype Responsive to Ruxolitinib. *Journal of Clinical Immunology* (2019) 39(8):776-85. doi: 10.1007/s10875-019-00687-4.

84. Baghad B, Benhsaien I, El Fatoiki FZ, Migaud M, Puel A, Chiheb S, et al. Chronic mucocutaneous candidiasis with STAT1 gain-of-function mutation associated with herpes virus and mycobacterial infections. *Annales de Dermatologie et de Venereologie* (2019). doi: 10.1016/j.annder.2019.09.597.

85. Carey B, Lambourne J, Porter S, Hodgson T. Chronic mucocutaneous candidiasis due to gain-of-function mutation in STAT1. *Oral Diseases* (2019) 25(3):684-92. doi: 10.1111/odi.12881.

86. Chen X, Xu Q, Li X, Wang L, Yang L, Chen Z, et al. Molecular and Phenotypic Characterization of Nine Patients with STAT1 GOF Mutations in China. *Journal of Clinical Immunology* (2019). doi: 10.1007/s10875-019-00688-3.

87. Gan C, Xu H. Clinical characteristics and biochemical immunoassay of chronic mucocutaneous candidiasis due to gain-of-function mutations in STAT1 in 5 children. *Journal of Chinical Pediatrics* (2019) 37(10):739-43. PubMed PMID: CSCD:6597416.

88. Henrickson SE, Dolan JG, Forbes LR, Vargas-Hernandez A, Nishimura S, Okada S, et al. Gain-of-function STAT1 mutation with familial lymphadenopathy and Hodgkin Lymphoma. *Frontiers in Pediatrics* (2019) 7(APR). doi: 10.3389/fped.2019.00160.

89. Kaleviste E, Saare M, Leahy TR, Bondet V, Duffy D, Mogensen TH, et al. Interferon signature in patients with STAT1 gain-of-function mutation is epigenetically determined. *Eur J Immunol* (2019) 49(5):790-800. Epub 2019/02/26. doi: 10.1002/eji.201847955. PubMed PMID: 30801692.

90. Kiykim A, Charbonnier LM, Akcay A, Karakoc-Aydiner E, Ozen A, Ozturk G, et al. Hematopoietic Stem Cell Transplantation in Patients with Heterozygous STAT1 Gain-of-Function Mutation. *Journal of Clinical Immunology* (2019) 39(1):37-44. doi: 10.1007/s10875-018-0575-y.

91. Lee JS, An Y, Yoon CJ, Kim JY, Kim KH, Freeman AF, et al. Germline gain-of-function mutation of STAT1 rescued by somatic mosaicism in immune dysregulation-polyendocrinopathy-enteropathy-X-linked-like disorder. *The Journal of allergy and clinical immunology* (2019). doi: 10.1016/j.jaci.2019.11.028.

92. Maeshima K, Ishii K, Shibata H. An adult fatal case with a STAT1 gain-of-function mutation associated with multiple autoimmune diseases. *Journal of Rheumatology* (2019) 46(3):325-7. doi: 10.3899/jrheum.180210.

93. Molho-Pessach V, Meltser A, Kamshov A, Ramot Y, Zlotogorski A. STAT1 gain-of-function and chronic demodicosis. *Pediatric Dermatology* (2019). doi: 10.1111/pde.14011.

94. Papadopoulou C, Omoyinmi E, Standing A, Pain CE, Booth C, D'Arco F, et al. Monogenic mimics of Behçet's disease in the young. *Rheumatology (United Kingdom)* (2019) 58(7):1227-38. doi: 10.1093/rheumatology/key445.

95. Renoux MC, Moreau J, Vigue MG. Chronic Lung Suppurative Disease in a Child Related to a STAT1 Heterozygous Gain-of-Function Mutation. *Archivos de bronconeumologia* (2019). doi: 10.1016/j.arbres.2019.10.014.

96. Rudilla F, Franco-Jarava C, Martínez-Gallo M, Garcia-Prat M, Martín-Nalda A, Rivière J, et al. Expanding the Clinical and Genetic Spectra of Primary Immunodeficiency-Related Disorders With Clinical Exome Sequencing: Expected and Unexpected Findings. *Frontiers in Immunology* (2019) 10. doi: 10.3389/fimmu.2019.02325.

97. Stellacci E, Moneta GM, Bruselles A, Barresi S, Pizzi S, Torre G, et al. The activating p.Ser466Arg change in STAT1 causes a peculiar phenotype with features of interferonopathies. *Clinical Genetics* (2019). doi: 10.1111/cge.13632.

98. Tirosh I, Spielman S, Barel O, Ram R, Stauber T, Paret G, et al. Whole exome sequencing in childhood-onset lupus frequently detects single gene etiologies. *Pediatric Rheumatology* (2019) 17(1). doi: 10.1186/s12969-019-0349-y.

99. van Zelm MC, Bosco JJ, Aui PM, De Jong S, Hore-Lacy F, O'Hehir RE, et al. Impaired STAT3-Dependent Upregulation of IL2Rα in B Cells of a Patient With a STAT1 Gain-of-Function Mutation. *Frontiers in immunology* (2019) 10:768. doi: 10.3389/fimmu.2019.00768.

100. Zimmerman O, Olbrich P, Freeman AF, Rosen LB, Uzel G, Zerbe CS, et al. STAT1 gain-of-function mutations cause high total STAT1 levels with normal dephosphorylation. *Frontiers in Immunology* (2019) 10(JULY). doi: 10.3389/fimmu.2019.01433.

101. Dupuis S, Dargemont C, Fieschi C, Thomassin N, Rosenzweig S, Harris J, et al. Impairment of mycobacterial but not viral immunity by a germline human STAT1 mutation. *Science* (2001) 293(5528):300-3. Epub 2001/07/14. doi: 10.1126/science.1061154. PubMed PMID: 11452125.

102. Dupuis S, Jouanguy E, Al-Hajjar S, Fieschi C, Al-Mohsen IZ, Al-Jumaah S, et al. Impaired response to interferon-alpha/beta and lethal viral disease in human STAT1 deficiency. *Nat Genet* (2003) 33(3):388-91. Epub 2003/02/19. doi: 10.1038/ng1097. PubMed PMID: 12590259.

103. Chapgier A, Boisson-Dupuis S, Jouanguy E, Vogt G, Feinberg J, Prochnicka-Chalufour A, et al. Novel STAT1 alleles in otherwise healthy patients with mycobacterial disease. *Plos Genetics* (2006) 2(8):1193-206. doi: 10.1371/journal.pgen.0020131. PubMed PMID: WOS:000240006900009.

104. Chapgier A, Wynn RF, Jouanguy E, Filipe-Santos O, Zhang S, Feinberg J, et al. Human complete Stat-1 deficiency is associated with defective type I and IIIFN responses in vitro but immunity to some low virulence viruses in vivo. *Journal of Immunology* (2006) 176(8):5078-83. doi: 10.4049/jimmunol.176.8.5078. PubMed PMID: WOS:000238769000071.

105. Chapgier A, Kong XF, Boisson-Dupuis S, Jouanguy E, Averbuch D, Feinberg J, et al. A partial form of recessive STAT1 deficiency in humans. *Journal of Clinical Investigation* (2009) 119(6):1502-14. doi: 10.1172/JCI37083.

106. Kong X-F, Ciancanelli M, Al-Hajjar S, Alsina L, Zumwalt T, Bustamante J, et al. A novel form of human STAT1 deficiency impairing early but not late responses to interferons. *Blood* (2010) 116(26):5895-906. doi: 10.1182/blood-2010-04-280586. PubMed PMID: WOS:000285583400020.

107. Averbuch D, Chapgier A, Boisson-Dupuis S, Casanova JL, Engelhard D. The clinical spectrum of patients with deficiency of signal transducer and activator of transcription-1. *Pediatric Infectious Disease Journal* (2011) 30(4):352-5. doi: 10.1097/INF.0b013e3181fdff4a.

108. Kristensen IA, Veirum JE, Moller BK, Christiansen M. Novel STAT1 Alleles in a Patient with Impaired Resistance to Mycobacteria. *Journal of Clinical Immunology* (2011) 31(2):265-71. doi: 10.1007/s10875-010-9480-8. PubMed PMID: WOS:000291169900014.

109. Vairo D, Tassone L, Tabellini G, Tamassia N, Gasperini S, Bazzoni F, et al. Severe impairment of IFN-gamma and IFN-alpha responses in cells of a patient with a novel STAT1 splicing mutation. *Blood* (2011) 118(7):1806-17. doi: 10.1182/blood-2011-01-330571. PubMed PMID: WOS:000294011500020.

110. Boisson-Dupuis S, Kong X-F, Okada S, Cypowyj S, Puel A, Abel L, et al. Inborn errors of human STAT1: allelic heterogeneity governs the diversity of immunological and infectious phenotypes. *Current Opinion in Immunology* (2012) 24(4):364-78. doi: 10.1016/j.coi.2012.04.011. PubMed PMID: WOS:000308898700002.

111. Sampaio EP, Bax HI, Hsu AP, Kristosturyan E, Pechacek J, Chandrasekaran P, et al. A novel STAT1 mutation associated with disseminated mycobacterial disease. *Journal of Clinical Immunology* (2012) 32(4):681-9. doi: 10.1007/s10875-012-9659-2.

112. Tsumura M, Okada S, Sakai H, Yasunaga S, Ohtsubo M, Murata T, et al. Dominant-negative STAT1 SH2 domain mutations in unrelated patients with mendelian susceptibility to mycobacterial disease. *Human Mutation* (2012) 33(9):1377-87. doi: 10.1002/humu.22113.

113. Hirata O, Okada S, Tsumura M, Kagawa R, Miki M, Kawaguchi H, et al. Heterozygosity for the Y701C STAT1 mutation in a multiplex kindred with multifocal osteomyelitis. *Haematologica* (2013) 98(10):1641-9. doi: 10.3324/haematol.2013.083741. PubMed PMID: WOS:000328543400026.

114. Shamriz O, Engelhard D, Rajs AP, Kaidar-Shwartz H, Casanova JL, Averbuch D. Mycobacterium szulgai chronic multifocal osteomyelitis in an adolescent with inherited STAT1 deficiency. *Pediatric Infectious Disease Journal* (2013) 32(12):1345-7. doi: 10.1097/01.inf.0000437067.43859.4c.

115. Boudjemaa S, Dainese L, Heritier S, Masserot C, Hachemane S, Casanova JL, et al. Disseminated BCG osteomyelitis related to STAT 1 gene deficiency mimicking a metastatic neuroblastoma. *Pediatric and developmental pathology : the official journal of the Society for Pediatric Pathology and the Paediatric Pathology Society* (2016). Epub 2016/03/29. doi: 10.2350/16-02-1778-cr.1. PubMed PMID: 27018766.

116. Burns C, Cheung A, Stark Z, Choo S, Downie L, White S, et al. A novel presentation of homozygous loss-of-function STAT-1 mutation in an infant with hyperinflammation—A case report and review of the literature. *Journal of Allergy and Clinical Immunology: In Practice* (2016) 4(4):777-9. doi: 10.1016/j.jaip.2016.02.015.

117. Muriel-Vizcaino R, Yamazaki-Nakashimada M, Lopez-Herrera G, Santos-Argumedo L, Ramirez-Alejo N. Hemophagocytic Lymphohistiocytosis as a Complication in Patients with MSMD. *Journal of Clinical Immunology* (2016) 36(5):420-2. doi: 10.1007/s10875-016-0292-3. PubMed PMID: WOS:000377370100003.

118. Naviglio S, Soncini E, Vairo D, Lanfranchi A, Badolato R, Porta F. Long-Term Survival After Hematopoietic Stem Cell Transplantation for Complete STAT1 Deficiency. *Journal of Clinical Immunology* (2017) 37(7):701-6. doi: 10.1007/s10875-017-0430-6.

119. Ueki M, Yamada M, Ito K, Tozawa Y, Morino S, Horikoshi Y, et al. A heterozygous dominant-negative mutation in the coiled-coil domain of STAT1 is the cause of autosomal-dominant Mendelian susceptibility to mycobacterial diseases. *Clin Immunol* (2017) 174:24-31. Epub 2016/11/20. doi: 10.1016/j.clim.2016.11.004. PubMed PMID: 27856304.

120. Averbuch D, Safadi R, Dar D, Wolf D, Cherniak M, Sorek R, et al. Successful Brincidofovir Treatment of Metagenomics-detected Adenovirus Infection in a Severely Ill Signal Transducer and Activator of Transcription-1-deficient Patient. *Pediatric Infectious Disease Journal* (2019) 38(3):297-9. doi: 10.1097/INF.0000000000002090. PubMed Central PMCID: PMCChimerix(United States).

121. Ying W, Liu D, Dong X, Wang W, Hui X, Hou J, et al. Current Status of the Management of Mendelian Susceptibility to Mycobacterial Disease in Mainland China. *Journal of Clinical Immunology* (2019) 39(6):600-10. doi: 10.1007/s10875-019-00672-x.

122. Sharfe N, Nahum A, Newell A, Dadi H, Ngan B, Pereira SL, et al. Fatal combined immunodeficiency associated with heterozygous mutation in STAT1. *Journal of Allergy and Clinical Immunology* (2014) 133(3):807-17. doi: 10.1016/j.jaci.2013.09.032.

123. Grunebaum E, Kim VH, Somers GR, Shammas A, Roifman CM. Bone marrow transplantation for monoallelic signal transducer and activator of transcription 1 deficiency. *J Allergy Clin Immunol* (2016) 138(2):612-5.e1. Epub 2016/04/12. doi: 10.1016/j.jaci.2016.02.009. PubMed PMID: 27061251.

124. Thoenia C, Hamiltona EA, Elkadria A, Murchiea R, Fiedlera K, Ovadiad A, et al. The effects of STAT1 dysfunction on the gut. *LymphoSign Journal* (2016) 3(1):19-33. doi: 10.14785/lpsn-2015-0012.

125. Hosking LM, Quach A, Slade CA, Galea MA, Richards S, Choo S, et al. Proceed with Caution: STAT1 GOF Diagnosis Missed Due to Intronic SNP. *J Clin Immunol* (2020) 40(3):547-50. Epub 2020/03/09. doi: 10.1007/s10875-020-00768-9. PubMed PMID: 32146551.

126. Marinelli L, Ristagno E, Fischer P, Abraham R, Joshi A. Cryptococcal pneumonia in an adolescent with a gain-of-function variant in signal transduction and activator of transcription 1 (STAT1). *BMJ Case Rep* (2020) 13(4). Epub 2020/04/25. doi: 10.1136/bcr-2019-234120. PubMed PMID: 32327459; PubMed Central PMCID: PMCPMC7202788.

127. Moriya K, Suzuki T, Uchida N, Nakano T, Katayama S, Irie M, et al. Ruxolitinib treatment of a patient with steroid-dependent severe autoimmunity due to STAT1 gain-of-function mutation. *Int J Hematol* (2020). Epub 2020/03/18. doi: 10.1007/s12185-020-02860-7. PubMed PMID: 32180118.

128. Renoux MC, Moreau J, Vigue MG. Chronic Lung Suppurative Disease in a Child Related to a STAT1 Heterozygous Gain-of-Function Mutation. *Arch Bronconeumol* (2020) 56(4):263-4. Epub 2019/11/27. doi: 10.1016/j.arbres.2019.10.014. PubMed PMID: 31767209.

129. Sáez-de-Ocariz M, Suárez-Gutiérrez M, Migaud M, O′Farrill-Romanillos P, Casanova JL, Segura-Mendez NH, et al. Rosacea as a striking feature in family members with a STAT1 gain-of-function mutation. *Journal of the European Academy of Dermatology and Venereology* (2020). doi: 10.1111/jdv.16241. PubMed Central PMCID: PMC31991004.

130. Sanghvi R, Siddik D, Hullah E, Shah T, Carey B. Chronic mucocutaneous candidiasis: a rare diagnosis in paediatric dentistry. *The British journal of oral & maxillofacial surgery* (2020). Epub 2020/05/01. doi: 10.1016/j.bjoms.2020.03.025. PubMed PMID: 32349899.

131. Zhang MR, Zhao F, Wang S, Lv S, Mou Y, Yao CL, et al. Molecular mechanism of azoles resistant Candida albicans in a patient with chronic mucocutaneous candidiasis. *BMC Infect Dis* (2020) 20(1):126. Epub 2020/02/13. doi: 10.1186/s12879-020-4856-8. PubMed PMID: 32046674; PubMed Central PMCID: PMCPMC7014776.

132. Zhu Y, Li L, Mao GS, Zhang L, Wang J, Li NN. Gene analysis of seven cases of primary immunodeficiency. *Translational Pediatrics* (2020) 9(2):117-25. doi: 10.21037/tp.2020.03.07. PubMed PMID: WOS:000528336600005.
